# Multiscale 3D Whole Joint Cellular and Molecular Mapping Reveals Disease‐Specific Neurovascular Plasticity Underlying the Structure‐Pain Relationship

**DOI:** 10.1002/advs.202511226

**Published:** 2025-11-06

**Authors:** Peng Chen, Jiaxin Chai, Abirami Soundararajan, R. Glenn Hepfer, Benjamin Kheyfets, Jiaxin Hu, Ishraq Alshanqiti, Swarnalakshmi Raman, Ikue Tosa, Jun Tae Huh, Matthew Yee, Brooke J. Damon, Shangping Wang, Yu Shin Kim, Man‐Kyo Chung, Mildred C. Embree, Janice S. Lee, Tong Ye, Hai Yao

**Affiliations:** ^1^ Department of Bioengineering Clemson University Clemson SC 29634 USA; ^2^ Department of Oral Health Sciences Medical University of South Carolina Charleston SC 29425 USA; ^3^ College of Dental Medicine Columbia University Irving Medical Center New York NY 10032 USA; ^4^ Columbia Stem Cell Initiative Columbia University Irving Medical Center New York NY 10032 USA; ^5^ Department of Neural and Pain Sciences School of Dentistry Center to Advance Chronic Pain Research University of Maryland Baltimore MD 21201 USA; ^6^ Department of Basic and Clinical Sciences School of Dentistry Umm Al‐Qura University Makkah 24382 Saudi Arabia; ^7^ Department of Oral and Maxillofacial Surgery School of Dentistry University of Texas Health Science Center at San Antonio San Antonio TX 78229 USA; ^8^ National Institute of Dental and Craniofacial Research (NIDCR) NIH Bethesda MD 20892 USA

**Keywords:** blood vessels, light sheet imaging, musculoskeletal joint, nerves, pain, structure, tissue clearing

## Abstract

Understanding musculoskeletal joints from a 3D multiscale perspective, from molecular to anatomical levels, is essential for resolving the confounding relationships between structure and pain, elucidating mechanisms regulating joint health and diseases, and developing new treatment strategies. Here, a musculoskeletal joint immunostaining and clearing (MUSIC) method specifically developed to overcome key challenges of immunostaining and optical clearing of intact joints are introduced. Coupled with large‐field light sheet microscopy, this approach achieves 3D high‐resolution, microscale neurovascular mapping within the context of whole‐joint anatomy without the need for image coregistration across various joints, including temporomandibular joints, knees, and spines, and multiple species, including mouse, rat, and pig. These results reveal 3D heterogeneous neurovascular distributions and previously uncharacterized neurovascular pathways within joints. Using two complementary models of joint disease, degeneration and injury, disease‐specific microscale neurovascular alterations are identified. These findings extend beyond conventional macroscale assessments of joint morphology and provide a framework to link structural changes with pain. Importantly, our results show that the relationship between joint structure and pain is not universal but disease‐dependent, underscoring distinct pain mechanisms in different disease contexts. This platform offers a powerful tool for multiscale 3D analysis, advancing understanding of joint pathophysiology and intricate interplay among joint tissues.

## Graphical abstract

This study presents MUSIC, a novel immunostaining and clearing method coupled with large‐field light sheet microscopy, enabling 3D high‐resolution cellular and molecular mapping of intact musculoskeletal joints across species and joint types. Using joint degenerative and injury models, disease‐specific neurovascular alterations are uncovered, revealing the context‐dependent relationship between joint structure and pain and advancing understanding of joint pathophysiology.

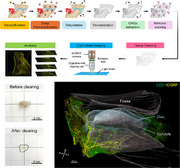

## 1 Introduction

Musculoskeletal diseases, such as temporomandibular joint disorders, osteoarthritis, and back pain, are the leading cause of disability, affecting ≈1.7 billion people worldwide and resulting in an estimated annual healthcare cost of $980 billion in the United States alone.^[^ ^1^, ^2^ ^]^ The musculoskeletal joint is a diverse and multiscale system comprising an array of different joint tissues (i.e., bones, connective tissues, and muscles), each with unique 3D structural and functional characteristics that span from the anatomical to molecular levels.^[^ ^3^, ^4^ ^]^ Dysregulation of one or multiple joint components at either the micro‐ or macroscale can initiate and drive disease onset and progression, leading to structural degradation, functional impairment, and pain. However, a major barrier to advancing our understanding of joint pathophysiology lies in the limited capacity of current methodologies to quantitatively and comprehensively capture structural alterations and disease features across scales. For instance, while magnetic resonance imaging (MRI) and computed tomography (CT) provide valuable macroscale anatomical information, they offer limited insight into cellular and molecular changes. Conversely, conventional histological approaches provide high‐resolution information at tissue and cellular levels but lack 3D spatial context at the organ scale. Investigating joint diseases from a single scale alone can be misleading. This limitation is exemplified by the long‐standing challenge of correlating joint structural changes with dysfunction and pain.^[^ ^5^, ^6^ ^]^ Radiographic evidence of joint abnormalities often shows poor concordance with patient‐reported pain, complicating diagnosis and impeding the development of effective therapies.^[^ ^5^, ^6^ ^]^ This discordance likely stems from a lack of microscale cellular and molecular information, highlighting the urgent need for integrated, multiscale approaches to musculoskeletal research.

Recent advancements in imaging have explored multiscale visualization of 3D joint vasculature by integrating MRI, CT, and optical imaging.^[^ ^7^ ^]^ While promising, this approach necessitates multiple scans and complex image coregistration across modalities, and it remains primarily limited to vascular mapping due to its dependence on vessel‐based contrast agents.^[^ ^7^ ^]^ Although the vascular system is vital for maintaining joint integrity and homeostasis by facilitating oxygen and nutrient delivery, molecular signaling, and cellular trafficking,^[^ ^8^, ^9^ ^]^ understanding the relationship between joint structure and pain necessitates the 3D mapping of additional cellular and molecular components, particularly the neural network.^[^ ^10^, ^11^ ^]^ Nerve fibers innervate the joints to sense the intrinsic and environmental stimuli (e.g., proprioceptive and noxious stimuli), transmit the signals to the brain where pain is perceived, and trigger somatic responses.^[^ ^11^ ^]^ Beyond their sensory role, neural elements also contribute to joint development and remodeling through the release of a variety of neurotrophic and regulatory factors.^[^ ^12^ ^]^ Therefore, there is a critical need for next‐generation 3D imaging techniques with improved molecular specificity that can capture the multiscale interplay among vascular, neural, and other tissue‐specific elements.

Optical imaging methods, especially fluorescence‐based ones, allow high‐resolution characterization of various cell and tissue structures owing to the availability of many molecular‐specific endogenous and exogenous fluorescent probes. However, 3D imaging large or thick samples presents a significant challenge due to the limited light penetration caused by tissue scattering, especially along the optical axial dimension. While multiphoton imaging can extend imaging depth to hundreds of micrometers,^[^ ^13^ ^]^ it remains insufficient for multiscale 3D whole‐joint imaging. To overcome these limitations, two primary strategies have been employed: serial section reconstruction and tissue clearing. In the serial section reconstruction, the sample is physically cut into a series of thin sections and imaged section by section for 3D reconstruction; this method is labor‐intensive, technique sophisticated, and subject to sample distortion due to the invasive sample sectioning.^[^ ^14^, ^15^, ^16^, ^17^ ^]^ Tissue clearing techniques offer a powerful alternative by rendering tissues optically transparent, thereby preserving their native 3D architecture and allowing for deeper and multiscale imaging.^[^ ^18^, ^19^ ^]^ Over the past decade, many tissue clearing protocols and methods have been developed,^[^ ^19^ ^]^ with the majority applied in the brain and increasing applications in other organs, including musculoskeletal tissues.^[^ ^20^, ^21^, ^22^, ^23^, ^24^, ^25^, ^26^ ^]^ However, previous reports only examined single joint components in mice, such as muscles^[^ ^21^, ^27^ ^]^ or bones.^[^ ^20^, ^22^, ^23^, ^24^, ^25^, ^28^, ^29^ ^]^ Systematic whole joint mapping presents significant challenges due to the complex architecture of musculoskeletal joints.^[^ ^30^ ^]^ Firstly, unlike the brain, which contains only soft tissues, musculoskeletal joints uniquely have hard and soft components, each exhibiting unique properties such as structure, composition, and optical transparency.^[^ ^30^ ^]^ Secondly, tissues in the joint, like the tendon and meniscus, are very dense with high collagen and glycosaminoglycan (GAG) contents, posing difficulties for antibody labeling and tissue clearing for the whole joint, as reported in recent publications.^[^ ^30^, ^31^ ^]^ Thirdly, the diverse array of joint types, including knee joints, spines, and temporomandibular joints (TMJs), necessitates a flexible approach accommodating different anatomical structures. Lastly, the size variability of joints in commonly utilized preclinical models, including mice, rats, and pigs, ranges from millimeters to centimeters, which underscores the need for scalable tissue clearing techniques and large‐field 3D imaging methods. A platform method with broad utility for 3D multiscale whole joint mapping across various joint types, sizes, and species has yet to be developed.

Here, we present a **mus**culoskeletal joint **i**mmunostaining and **c**learing technique (**MUSIC**), which specifically addresses key challenges of immunostaining and optical clearing of intact joints with various types and sizes through decalcification, deep joint permeabilization with small‐micelle detergent and GAGs extraction, and antibody stabilization. Coupled with fast and large‐field light sheet microscopy, our approach achieves 3D whole‐joint cellular and molecular mapping in a range of joints—including TMJs, knees, and spines—across multiple species, including mice, rats, and pigs. Our method allows multiscale 3D joint imaging of both macroscale joint anatomy and microscale cellular and molecular features, while no image coregistration is needed. Using the mouse TMJ as an example, we demonstrated successful immunostaining, clearing, and imaging of the 3D microscale neurovascular networks within the entire intact joint under healthy and diseased conditions. Our results illustrated the heterogeneous distribution of vessels and nerves in healthy TMJ and revealed detailed pain‐sensing nerve fiber innervation pathways. Using a well‐established mouse model of degenerative joint disease with global deficiency in proteoglycan 4 (*Prg4^‐/‐^*),^[^ ^32^, ^33^ ^]^ we revealed hallmark TMJ structure degeneration from a 3D perspective that was coupled with a drastically vascularized and innervated lateral capsule and synovial tissues and altered condyle vasculature networks not previously reported. In a TMJ injury mouse model, the forced‐mouth opening (FMO) model,^[^ ^34^ ^]^ we further characterized the temporal 3D plasticity of neurovascular structure and identified distinct microscale degenerative changes of pain‐sensing nerves within the injured anterior region of the TMJ disc, despite the absence of detectable macroscale morphological alterations. These disease‐specific microscale neurovascular alterations extended beyond conventional macroscale assessments of joint morphology and provided a framework to link structural changes with pain. Importantly, our results revealed that the relationship between joint structure and pain is not the same across disease conditions, underscoring disease‐dependent pain mechanisms. Additionally, we have demonstrated our method in mouse knees and found sprouting of 3D neurovascular structures in the knee capsules of *Prg4^‐/‐^* mice compared to wild‐type mice. Our method was further scaled up to map the 3D neurovascular structures in the whole rat TMJ, knee, and spine samples, and large pig TMJ discs with surrounding tissue attached. Overall, we provided a scalable and versatile tissue clearing and imaging method for multiscale 3D whole joint cellular and molecular mapping in musculoskeletal systems. This method allows us to quantitatively study both macroscale and microscale joint structure and neurovascular changes in diseases, which could help dissect the relationship between joint structure and pain alongside disease progression, repair, and following therapy, thereby advancing mechanistic studies and treatment development.

## 2 Results

### 2.1 MUSIC for Whole Joint Cellular and Molecular Mapping

**Figure 1:**
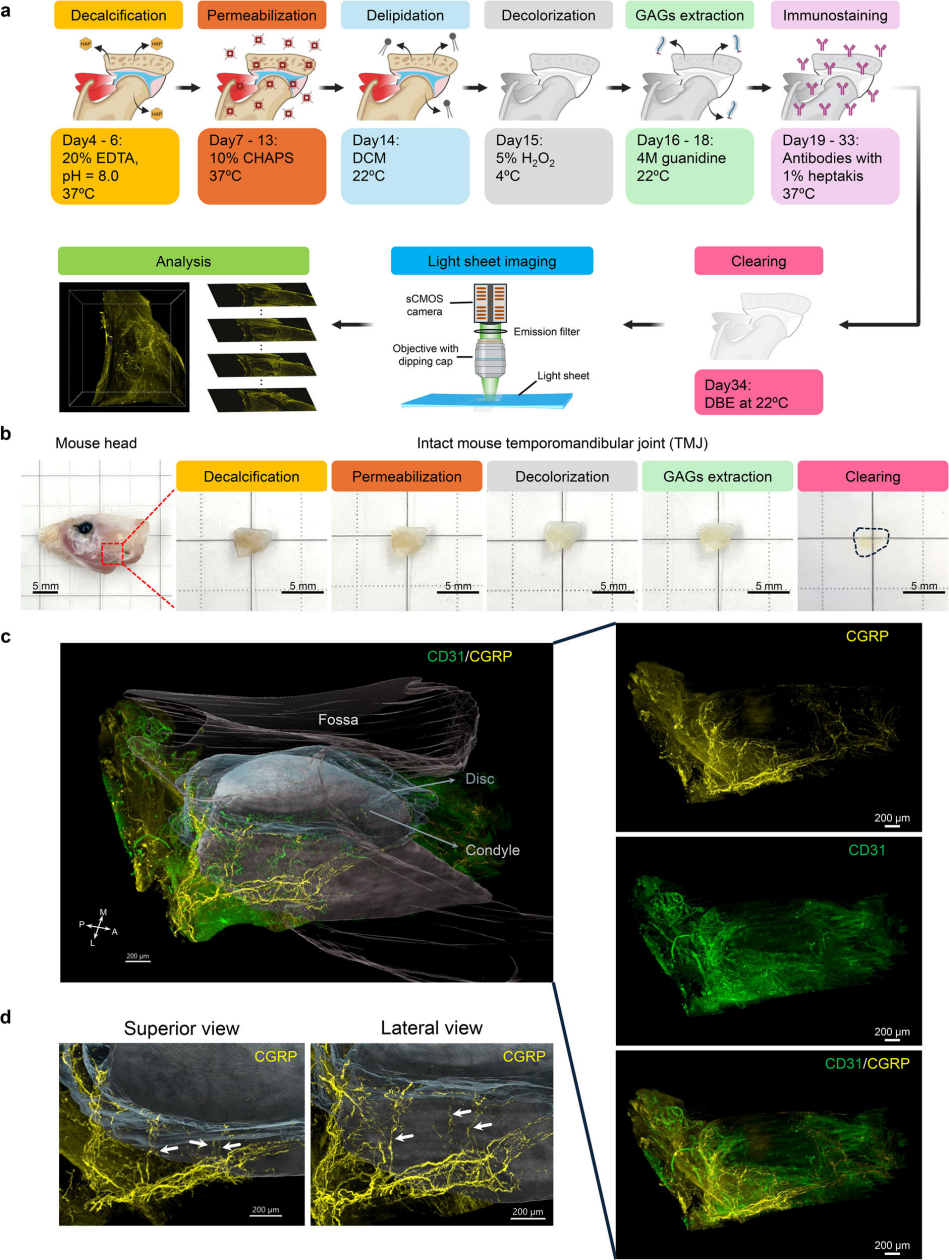
MUSIC for whole joint neurovascular mapping in mouse TMJ. a) Schematic workflow and timeline for the MUSIC method with mouse TMJ samples. Fixed joint samples were first decalcified in 20% EDTA solution and then incubated in 10% CHAPS solution for deep permeabilization. Then, they are dehydrated, delipidated with DCM, and decolored with 5% H_2_O_2_ solution. Next, the joint samples were treated with 4 m guanidine solution to extract the GAGs from the joint tissue extracellular matrix while maintaining their collagen backbone. Then, the joints were stained with primary and secondary antibodies (**Table** **S1**, Supporting Information) immunostaining solution with the addition of 1% heptakis(2,6‐di‐O‐methyl)‐β‐cyclodextrin to minimize antibody aggregation. Finally, the joints were cleared in DBE and imaged with a large‐field light sheet microscope. The 3D mapping data were further reconstructed and analyzed. Similar procedures were applied to other joints (**Table** **S2**, Supporting Information). b) Photos of mouse head and mouse TMJs along MUSIC procedures. The red dashed square highlighted the region where the TMJ was harvested. The black dashed line depicted the contour of a cleared mouse TMJ. Scale bar, 5 mm. c) Whole joint neurovascular mapping in a mouse TMJ. The TMJ condyle (including both the bone and overlying condylar cartilage), disc, and fossa were segmented, reconstructed, and rendered in grey, translucent light blue, and translucent light brown, respectively, to provide a joint coordinate to visualize the neurovascular structure distribution (Figure S3a,b, Supporting Information). 3D data from each imaging channel and merged channel without rendering were displayed on the right side. Signals from muscles were removed for visualization. Scale bar, 200 µm. d) Zoom‐in view at the lateral condyle neck region of the mouse TMJ from superior and lateral angles. White arrows highlighted the nerve strands branching out from the major nerve bundles and innervating the TMJ disc. Scale bar, 200 µm. A, anterior; P, posterior; M, medial; L, lateral.

To achieve whole joint immunostaining and clearing, a new pipeline was developed based on the unique joint features. First, the fixed joints were decalcified and then treated with a deep permeabilization chemical, CHAPS (3‐[(3‐cholamidopropyl) dimethylammonio]‐1‐propanesulfonate). CHAPS is a zwitterionic detergent that can form much smaller micelles than traditional tissue‐clearing detergents, like Triton X‐100, allowing rapid and efficient tissue permeabilization, as recently demonstrated with human brain tissues.^[^ ^31^ ^]^ Next, guanidine hydrochloride was used to remove the glycoproteins in joint tissues with high GAGs while keeping the fixed collagen backbones to achieve extracellular matrix loosening,^[^ ^31^ ^]^ enhancing the immunostaining antibody penetration. Given the various sizes of joints across different species, ranging from 5 mm to 5 cm, several days of antibody incubation are still needed, even with our efforts to deeply permeabilize the samples. Antibodies aggregate during the prolonged staining process, causing problems for labeling, visualization, and data analysis.^[^ ^35^ ^]^ To mitigate this problem, we used heptakis(2,6‐di‐O‐methyl)‐β‐cyclodextrin, a chemical that has been recently proven to reduce antibody aggregation.^[^ ^35^ ^]^ Finally, the organic solvent, dibenzyl ether (DBE), was selected as a refractive index matching agent for tissue clearing, given its superior compatibility with immunostaining and its excellent performance in clearing complicated organs, including musculoskeletal bones and muscles.^[^ ^28^, ^36^, ^37^, ^38^ ^]^ With optimizations based on the unique features of musculoskeletal joints, we finalized the MUSIC protocol for the whole joint mapping (**Figure** **1**a). For fast and large‐field imaging, an advanced light sheet microscope^[^ ^39^ ^]^ was applied for whole joint mapping (Figure 1a).

Using the mouse TMJ as an example, we demonstrated successful whole‐joint clearing using our method (Figure 1b). With the staining of validated CD31 and calcitonin gene‐related peptide (CGRP) antibodies for blood vessels and pain‐sensing nerve fibers, respectively (Figure S1 and Table S1, Supporting Information), we achieved neurovascular mapping of the entire intact mouse TMJ (Figure 1c). We further validated efficient whole joint immunostaining in mouse TMJs with the MUSIC method (Figure S2, Supporting Information). Our method inherently captures the macroscale joint anatomy, allowing easy spatial registration of individual joint components (Figure S3, Supporting Information). Through image segmentation, we can resolve the microscale neurovascular distribution in different joint tissues in the spatial context of the whole joint without the need of complicated image coregistration (Figure 1c; Figure S3a,b, and Movie S1, Supporting Information). Previous literature based on 2D histological analysis has reported innervation in the TMJ disc with inconsistent conclusions.^[^ ^40^ ^]^ Our results confirmed that no blood vessels and nerves are present in the central region of the disc (Figure 1c). High density of neurovascular structures was observed in the anterior and posterior regions of the disc. In the zoomed‐in view of the lateral side of the TMJ, strands of nerve fibers that branch from the major nerve bundles and innervate the disc were observed (Figure 1d), unveiling the microscopic level of the innervation pathway of the TMJ disc. With our method, we can access the neurovascular spatial pattern in the entire TMJ that has never been seen before. It provides an unprecedented opportunity to explore its spatial distributions, tissue‐specific features, and connections/interactions between each joint component.

### 2.2 Whole Joint Degenerative Changes in *Prg4^‐/‐^* Mouse TMJ

**Figure 2:**
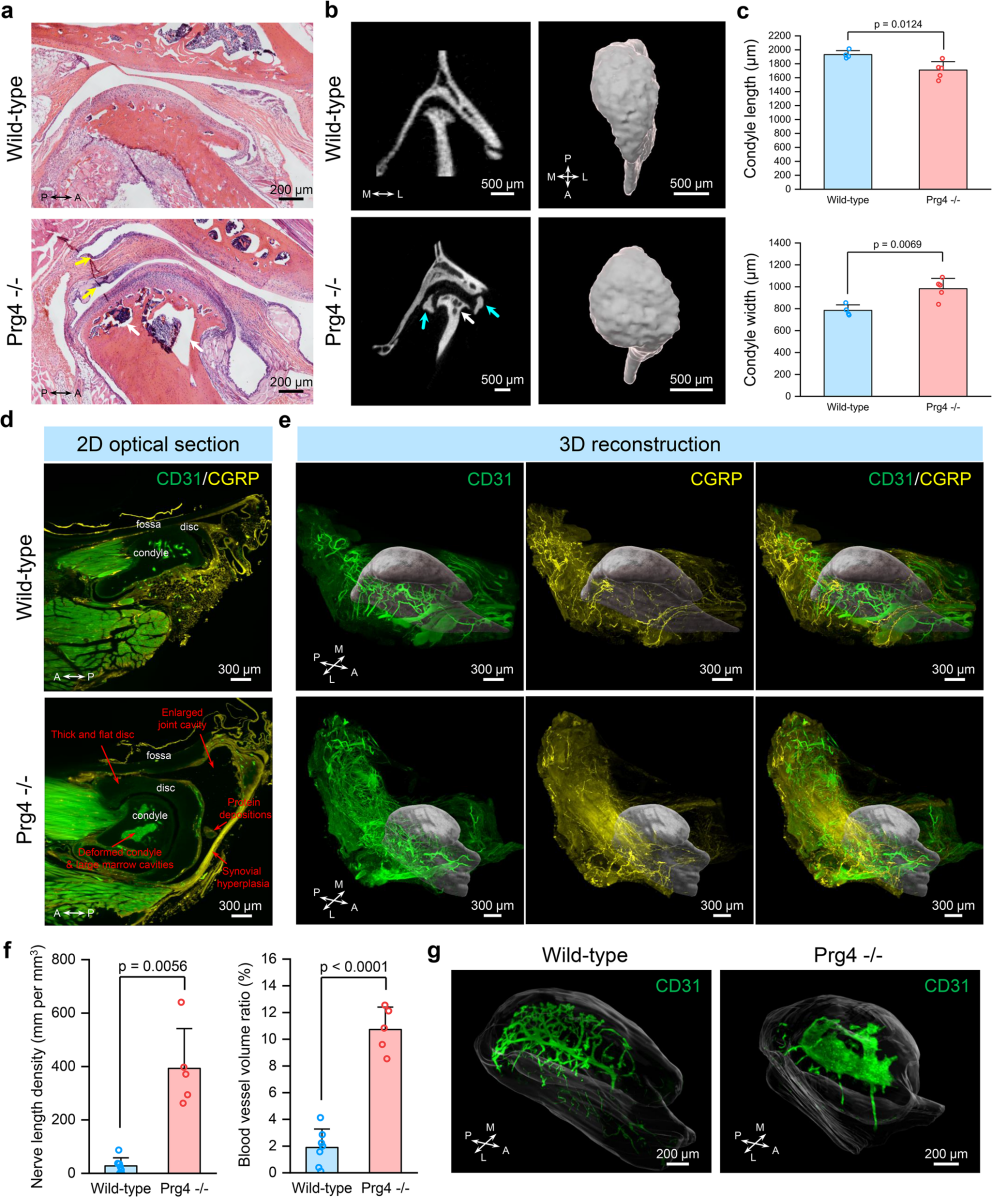
Whole joint neurovascular mapping in degenerative *Prg4^‐/‐^* mouse model. a) H&E staining results with mouse TMJs in wild‐type and *Prg4^‐/‐^* mice. White arrows highlighted the large marrow cavities in the TMJ condyle head, and yellow arrows pinpointed the inflamed synovial membrane in *Prg4^‐/‐^* mice. Scale bar, 200 µm. b) µCT imaging results with mouse TMJs in wild‐type and *Prg4^‐/‐^* mice. Left, 2D µCT images of mouse TMJ. White arrows highlighted the large marrow cavities in the TMJ condyle head, while the cyan arrows depicted the TMJ disc calcification in *Prg4^‐/‐^* mice. Right, 3D reconstruction of mouse TMJ condyle head. Scale bar, 500 µm. c) Quantitative TMJ condyle head size comparison between wild‐type (*n* = 4 joints from 4 mice) and *Prg4^‐/‐^* mice (*n* = 5 joints from 5 mice). *p*‐value was determined with a two‐sided *t*‐test. All data depict mean ± standard deviation. d) 2D optical section from the 3D neurovascular mapping in the whole mouse TMJ in wild‐type and *Prg4^‐/‐^* mice. TMJ condyle, disc, and fossa are labeled. The red arrows highlighted the pathological changes in the TMJ of *Prg4^‐/‐^* mice. Scale bar, 300 µm. e) 3D reconstruction results of whole joint neurovascular mapping in mouse TMJ of wild‐type and *Prg4^‐/‐^* mice. The TMJ condyles, including both the bone and overlying condylar cartilage, were segmented from the mapping data and rendered in grey to provide a spatial reference. Scale bar, 300 µm. f) Quantitative nerve length density and blood vessel volume ratio in wild‐type (*n* = 7 joints from 4 mice) and *Prg4^‐/‐^* TMJs (*n* = 5 joints from 3 mice). The nerve and vessel densities were quantified at the lateral capsule with synovial tissue (Figure S4a,b, Supporting Information). *p*‐value was determined by a two‐sided t‐test. All data depict mean ± standard deviation. g) 3D reconstruction of vascular structures within the TMJ condyle head. The TMJ condyle, including both the bone and overlying condylar cartilage, was segmented and rendered in translucent color to provide a spatial reference. Scale bar, 200 µm. A, anterior; P, posterior; M, medial; L, lateral.

Degenerative joint disease, such as osteoarthritis, is a major cause of musculoskeletal dysfunction and pain.^[^ ^41^ ^]^ Few studies have investigated the TMJ neurovascular structure in osteoarthritic joints, with inconsistent literature reports using 2D histological methods.^[^ ^42^ ^]^ Here, we applied our MUSIC method to investigate how the 3D neurovascular structure changes when joints degenerate. We used a well‐established genetic mouse model globally deficient in *Prg4*.^[^ ^32^, ^33^ ^]^ The *Prg4* gene encodes for the protein, lubricin, a critical glycoprotein in synovial joint fluid and cartilage superficial zone that provides joint lubrication.^[^ ^32^ ^]^ Mice lacking lubricin develop age‐dependent osteoarthritis‐like degeneration in joints, such as TMJs and knees.^[^ ^32^, ^33^, ^43^ ^]^ We first confirmed the joint degeneration in *Prg4^‐/‐^* mice through traditional hematoxylin and eosin (H&E) staining. The *Prg4^‐/‐^* TMJ showed a thickened disc, synovial membrane hyperplasia, articular cartilage erosion and fibrillation, and large marrow cavities, which matched well with literature observations (**Figure** **2**a).^[^ ^32^, ^33^, ^43^ ^]^ TMJ bone morphometric analysis performed with micro‐computed tomography (µCT) imaging showed that the *Prg4^‐/‐^* TMJ had calcified disc, deformed condyle, and condyle bone resorption (Figure 2b). The *Prg4^‐/‐^* TMJ condyle 3D shape was rounder with significantly decreased length and increased width compared to wild‐type mice (Figure 2b,c). We confirmed similar pathological changes using our MUSIC mapping method relative to traditional histologic analysis in the *Prg4^‐/‐^* TMJ from a single 2D optical section (Figure 2d). In addition, through 3D reconstruction and segmentation, we observed 3D condyle anatomical changes in the *Prg4^‐/‐^* TMJ as shown in the µCT analysis (Figure 2e). More importantly, unlike H&E and µCT techniques, the MUSIC protocol allowed us to map the microscale neurovascular structure in the whole joint by immunostaining CGRP (a marker of peptidergic sensory nerves) and CD31 (a marker of blood vessels) and uncover a 3D highly vascularized and innervated lateral capsule and synovial tissue in the *Prg4^‐/‐^* TMJ (Figure 2e,f; Figures S4a,b,S5a and Movie S2,S3, Supporting Information). The internal vasculature network can also be visualized with the integration of immunofluorescence and blood autofluorescence using the blood retention sample preparation approach (Figure 2g).^[^ ^44^ ^]^ The vasculature architecture dramatically changed within the *Prg4^‐/‐^* TMJ condyle head subchondral bone (Figure 2g; Movie S4,S5, Supporting Information), which may indicate aberrant subchondral bone remodeling. The pathways connecting the vasculature between the inner and outer regions of the condyle were also observed at the condyle neck (Figure S6, Supporting Information), which could provide new insights into the crosstalk among different joint components.

### 2.3 Spatiotemporal Neurovascular Remodeling in FMO Injury Mouse Model Links Structure and Pain

**Figure 3:**
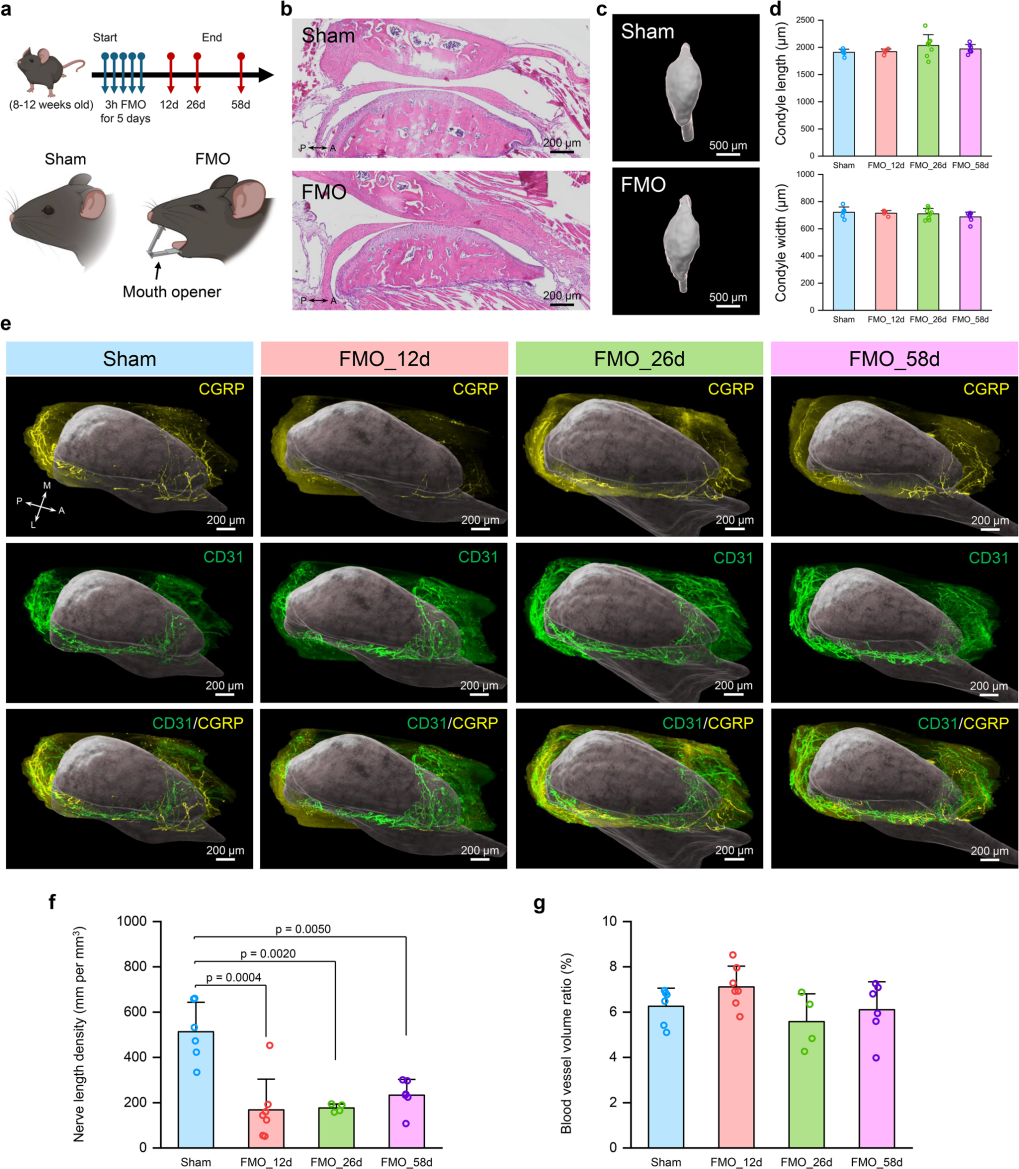
Whole joint neurovascular mapping in forced mouth opening (FMO) TMJ injury model. a) Schematic timeline for the FMO TMJ injury model. A customized mouth opener was applied between the maxillary and mandibular incisors to cause TMJ injury in the FMO group. b) H&E staining results with mouse TMJs in Sham and FMO mice at 58 days after the FMO procedure. Scale bar, 200 µm. c) 3D reconstruction of mouse TMJ condyle heads in Sham and FMO mice at 12 days after FMO procedure. Scale bar, 500 µm. d) Quantitative condyle size measurements based on condyle µCT 3D reconstruction with Sham (*n* = 6 joints from 6 mice), FMO_12d (*n* = 4 joints from 4 mice), FMO_26d (*n* = 8 joints from 8 mice), FMO_58d (*n* = 7 joints from 7 mice). e) 3D neurovascular mapping in the TMJ disc of Sham and FMO mice. The TMJ disc data was segmented from the whole joint mapping data. The TMJ condyles, including both the bone and overlying condylar cartilage, were reconstructed and rendered in grey to provide a spatial reference. Scale bar, 200 µm. f) Quantitative nerve length density at the anterior region of the TMJ disc in Sham (*n* = 6 joints from 4 mice), FMO_12d (*n* = 7 joints from 5 mice), FMO_26d (*n* = 4 joints from 2 mice), FMO_58d (*n* = 6 joints from 4 mice). *p*‐value was determined by one‐way ANOVA with Bonferroni post‐hoc test. g) Quantitative blood vessel volume ratio at the anterior region of the TMJ disc in Sham (*n* = 6 joints from 4 mice), FMO_12d (*n* = 7 joints from 5 mice), FMO_26d (*n* = 4 joints from 2 mice), FMO_58d (*n* = 6 joints from 4 mice). The nerve and vessel densities were quantified at the anterior region of the TMJ disc (Figure S4c,d, Supporting Information). All data depict mean ± standard deviation. A, anterior; P, posterior; M, medial; L, lateral.

TMJ is one of the most frequently used load‐bearing joints. Overloading the joint can cause joint injury, pain, and dysfunction.^[^ ^34^, ^45^ ^]^ TMJ injury animal models, such as the forced‐mouth opening (FMO) model, have been developed to investigate the joint's structural response to biomechanical stimulus and how it relates to pain and joint function.^[^ ^34^, ^45^ ^]^ With our whole joint mapping approach, we can holistically study this question. Adult mice (8–12 weeks old) were subjected to either FMO or Sham procedures under anesthesia for 3 h per day for five consecutive days, as described in previous publications (**Figure** **3**a).^[^ ^34^ ^]^ Sham mice were sacrificed 12 days after the procedure, while FMO mice were euthanized at multiple time points post‐injury (12 days (FMO_12d), 26 days (FMO_26d), and 58 days (FMO_58d)) (Figure 3a). Our recently published pain behavior data have demonstrated that FMO induces long‐last mechanical pain.^[^ ^34^ ^]^ However, histologically, we did not see apparent differences among groups in the cartilage, synovial, or condyle bone (Figure 3b). 3D µCT analysis of condyle size also showed no significant differences among the groups (Figure 3c,d). In contrast, time‐course neurovascular structure changes in the TMJ disc were observed through our 3D mapping data (Figure 3e; Movie S6–S9, Supporting Information). We quantified the nerve and blood vessel density in the anterior region of the TMJ disc (Figure S4c,d, Supporting Information), where tissue injury happened during the FMO procedure.^[^ ^46^, ^47^ ^]^ Our results showed that the nerve length and branch point density were significantly reduced following the injury and then gradually increased over time, even though it did not return to the same level as the Sham group (Figure 3f; Figure S5b, Supporting Information). Such neural changes indicate a dynamic process of nerve terminal degeneration and regeneration, similar to what has been reported in skin wound healing.^[^ ^48^ ^]^ These data may help explain our pain behavior results of sustained pain in FMO mice.^[^ ^34^ ^]^ It is possible that the remaining nerve fibers in the TMJ discs get sensitized after injury, and/or the nerve terminal injury causes neuropathic‐like persistent pain.^[^ ^47^, ^49^ ^]^ Although the pain sources in TMJ remain elusive, a more detailed investigation of neural changes in the TMJ should provide new insights into peripheral pain‐sensing mechanisms. Whole joint neural mapping provides the anatomic or structural basis for further investigation of joint pain. Interestingly, the blood vessel length, branch point, and volume density showed no significant differences among the groups (Figure 3g; Figure S5b, Supporting Information). The disparity between the neural and vascular structure changes suggests that vascularity shows higher resistance to tissue injury or blood vessel ingrowth preceding innervation during wound healing.^[^ ^50^ ^]^ Our data uncovered significant neurovascular changes before any apparent changes in joint anatomy (Figure 3b–d), demonstrating the sensitivity of our method to detect microscopic levels of cellular and molecular alterations. In addition, it has been controversial to correlate joint structure changes (i.e., joint morphology through CT or MRI) and joint symptoms (e.g., pain).^[^ ^6^ ^]^ Our results offer a new data dimension, providing microscopic‐level information from a whole joint perspective to deepen our understanding of the joint's structure‐pain relationship.

### 2.4 Whole Joint Neurovascular Mapping in Mouse Knees

**Figure 4:**
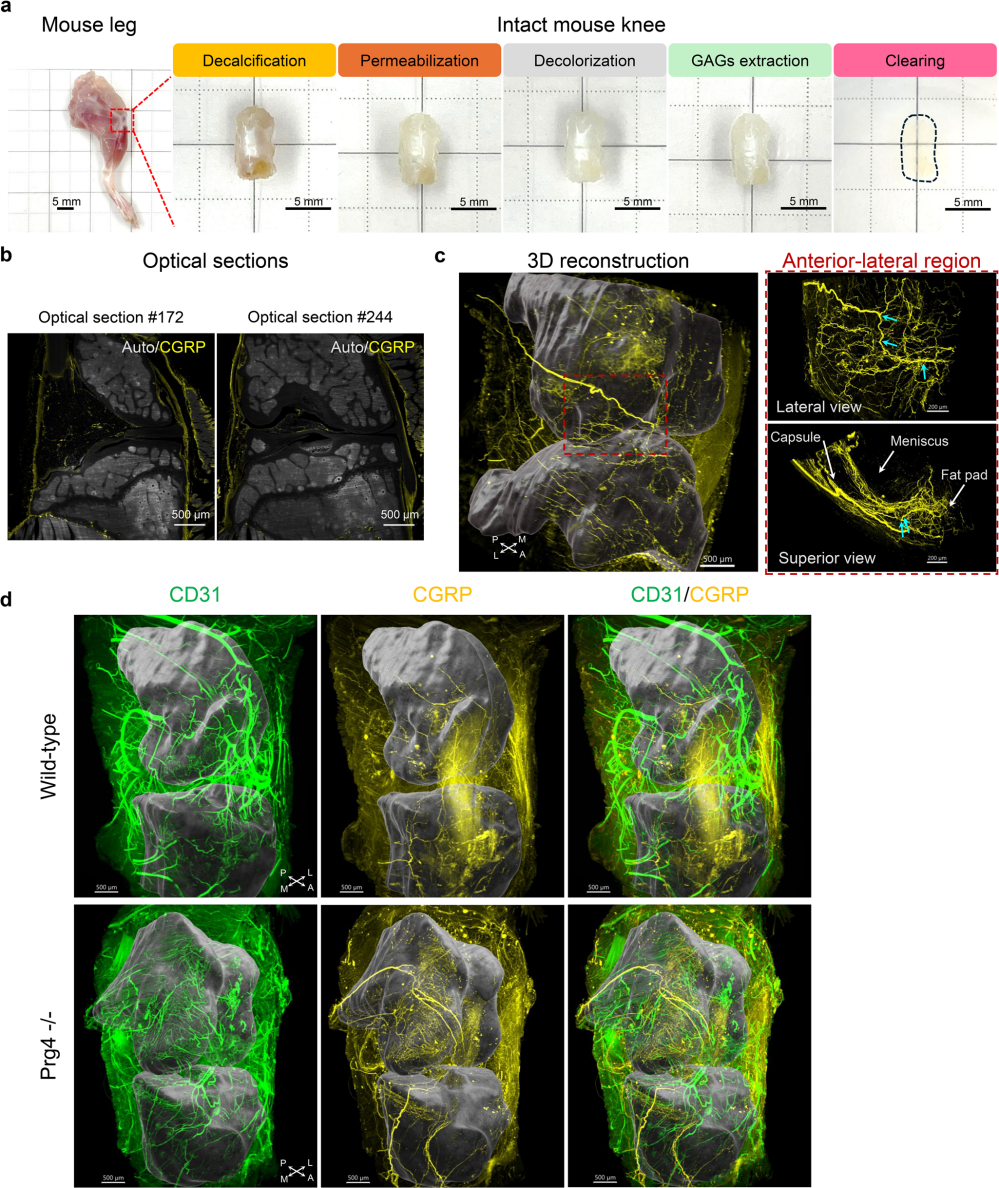
Whole joint neurovascular mapping in mouse knees. a) Photos of mouse leg and mouse knee along MUSIC procedures. The red dashed square highlighted the region where the mouse knee was harvested. The black dashed line depicted the contour of a cleared mouse knee. Scale bar, 5 mm. b) 2D optical sections from the 3D neural mapping in a mouse knee. Autofluorescence (Auto) signals were collected using a 488 nm laser and an emission filter of 525/50 nm. Scale bar, 500 µm. c) 3D reconstruction results of whole joint mapping in a mouse knee. Left, 3D whole joint reconstruction. The femur and tibia, including both the bone and overlying cartilage, were segmented and rendered in dark grey to provide a spatial reference (Figure S3c,d, Supporting Information). The red dashed square highlighted the lateral region of the mouse knee for high‐resolution imaging. Scale bar, 500 µm. Right, zoom‐in views at the lateral region of the mouse knee. Cyan arrows showed the nerves branching points. The joint capsule, meniscus, and fat pad were indicated with white arrows. Scale bar, 200 µm. d) 3D reconstruction results of whole joint mapping in mouse knees in wild‐type and *Prg4^‐/‐^* mice. The femur and tibia, including both the bone and overlying cartilage, were segmented and rendered in dark grey to provide a spatial reference. Scale bar, 500 µm. A, anterior; P, posterior; M, medial; L, lateral.

TMJs and knee joints are two major synovial joints in the body, sharing similarities and having intrinsic differences. To demonstrate the broad utility of our method, we applied the MUSIC protocol used for the mouse TMJs to mouse knees. We achieved successful tissue clearing of mouse knee joints (**Figure** **4**a) and labeled the pain‐sensing CGRP‐positive nerve fibers throughout the intact mouse knee (Figure 4b). From 2D optical sections collected at different knee locations, we can clearly see a high density of CGRP nerve distributions in the fat pad, capsule, and meniscus peripheral tissues (Figure 4b). Nerve fibers were also seen in the ligaments at the center of the knee, demonstrating the complete penetration of antibodies into the knee (Figure 4b). Beyond a glimpse of the knee joint innervation pattern through separate 2D optical sections, as done traditionally, we can now present the neural network in the entire knee through 3D reconstruction and trace the path of continuous long nerve fibers and their connectivity, as well as their spatial location and distributions within the joint anatomy (Figure 4c; Figure S3c,d, Supporting Information). Zoomed to the lateral region of the knee (highlighted in a dashed red square), nerves branched out from a major nerve bundle and innervated the fat pad, meniscus peripheral tissues, and capsule (Figure 4c), revealing the complex 3D neural network and connectivity among joint tissues that can hardly be detected with 2D methods. Moreover, we investigated how the neurovascular structure changes under degenerative conditions in the knee of the *Prg4^‐/‐^* mouse model. As reported in the literature, TMJs and knees developed osteoarthritis‐like degenerative changes in *Prg4^‐/‐^* mice.^[^ ^32^, ^43^ ^]^ Our results revealed an increased neurovascular density in the *Prg4^‐/‐^* mouse knee joint, confirming other studies (Figure 4d; Movie S10,S11, Supporting Information).^[^ ^32^, ^43^ ^]^ Benefiting from whole joint mapping, we can see how the neurovascular architectures change with dense nerves and vessels radiating through the *Prg4^‐/‐^* knee joint (Figure 4d). Our results demonstrate the application of our method within the broader orthopedic field, opening new opportunities to investigate knee joint diseases.

### 2.5 Scalable 3D Mapping in Joints of Larger Animal Species

**Figure 5:**
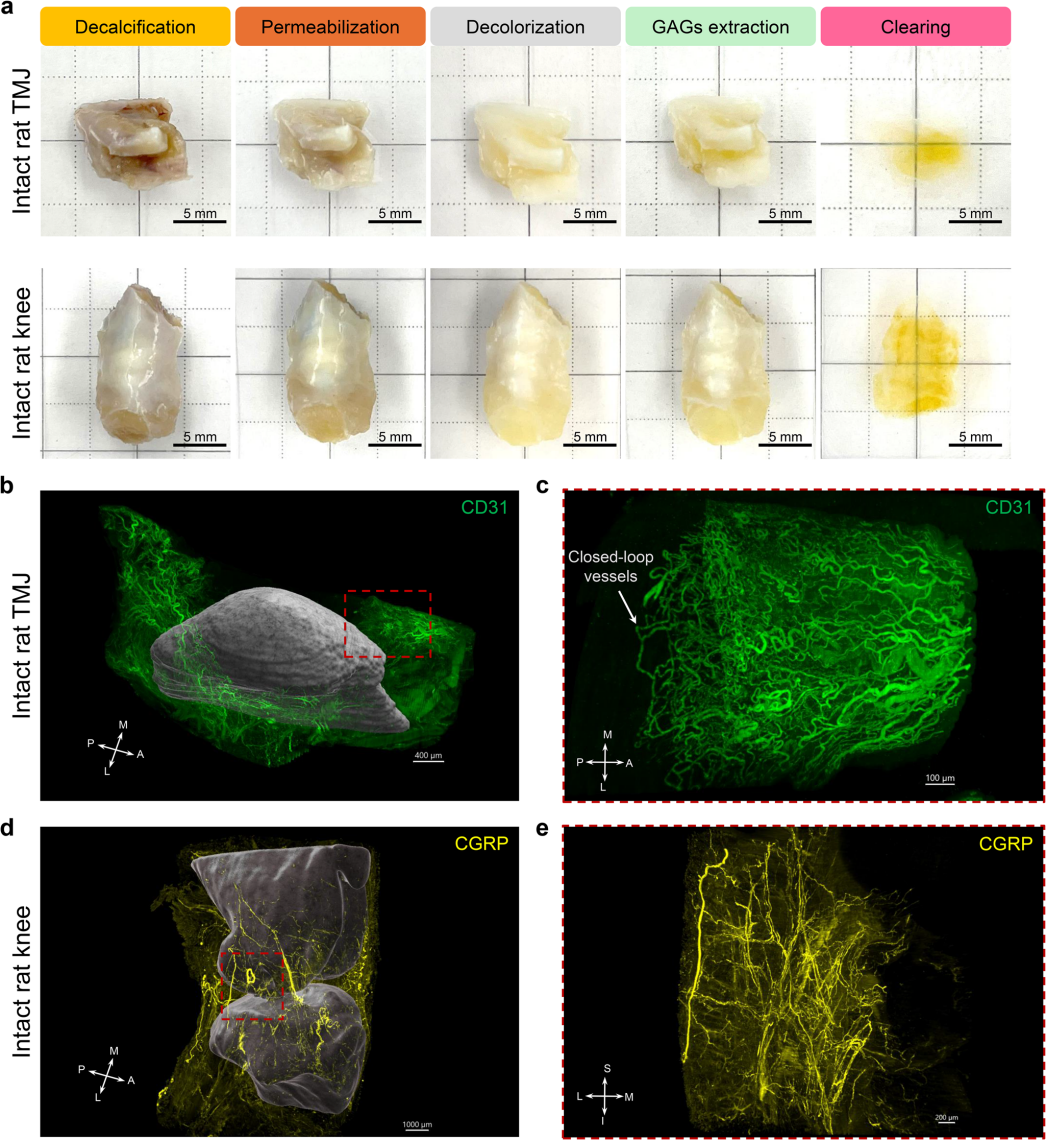
Scalable whole joint neurovascular mapping in rat joints. a) Photos of rat TMJs and knees along MUSIC procedures. Scale bar, 5 mm. b) 3D reconstruction results of whole joint vascular mapping in a rat TMJ. TMJ condyle, including both the bone and overlying condylar cartilage, was segmented and rendered in grey to provide a spatial reference. The red dashed square depicted the anterior region of the rat TMJ disc for high‐resolution imaging. Scale bar, 400 µm. c) Zoom‐in views of the anterior region of the rat TMJ. The white arrow highlighted the closed‐loop vessels at the edge of vascularization. Scale bar, 100 µm. d) 3D reconstruction results of whole joint neural mapping in a rat knee. Knee femur and tibia, including both the bone and overlying cartilage, were segmented and rendered in dark grey to provide a spatial reference. The red dashed square depicted the lateral region of the rat knee for high‐resolution imaging. Scale bar, 1000 µm. e) Zoom‐in views of the lateral region of the rat knee. Scale bar, 200 µm. A, anterior; P, posterior; M, medial; L, lateral; S, superior; I, inferior.

Various animal species have been used in musculoskeletal studies to pave the way for clinical research. Commonly used preclinical models include mice, rats, and pigs, with increasing joint dimensions as the animal size increases. 3D mapping of large joints presents critical challenges due to tissue clearing and immunostaining difficulties. Scalability issues were well considered when our method was initially developed. We strategically used CHAPS and guanidine treatments for deep permeabilization; these two chemicals are proven to perform excellently for large human tissues.^[^ ^31^ ^]^ Sufficient joint permeabilization allows effective tissue homogenization and antibody penetration to facilitate clearing and immunostaining of large samples. In addition, using heptakis(2,6‐di‐O‐methyl)‐β‐cyclodextrin minimizes the antibody aggregation issue during prolonged antibody incubation.^[^ ^35^ ^]^ We successfully cleared and mapped the rat TMJs (≈10 mm), knees (≈10 mm), and spine samples (≈7 mm), as well as pig TMJ discs with surrounding tissues (≈40 mm) (**Figure** **5**a; Figure S7, Supporting Information), demonstrating the scalability and versatility of our method for musculoskeletal applications. Our results on rat TMJs showed densely distributed blood vessels in the anterior and posterior regions of the TMJ disc (Figure 5b; Movie S12, Supporting Information), which agrees with the mouse data, supporting the conservation of neurovascular structures among species. Close‐looped vessels found at the border of the disc vasculature verified the absence of vessels in the disc center region (Figure 5c). Rat knee mapping data revealed a similar innervation pattern to the mouse knee, with high‐density innervation in the joint capsule and meniscus peripheral tissues (Figure 5d,e; Movie S13, Supporting Information).

In addition, we cleared and imaged the neural structure of the rat spine at the sacrum sections (Figure S7a,b, Supporting Information). High‐resolution imaging data revealed the pain‐sensing innervation at the intersection of two sacrum sections (Figure S7c and Movie S14, Supporting Information). Similarly, a large pig TMJ disc with surrounding tissues, with a size of 33.5 mm × 43.8 mm × 15.6 mm was also successfully immunostained and cleared. No nerves were observed at the disc central region, with fiber bundles at the lateral, anterior, and posterior regions (Figure S7d–f and Movie S15, Supporting Information). These results illustrated the scalability and versatility of our method, providing a platform method for musculoskeletal research.

## 3 Discussion

Understanding musculoskeletal joints from a multiscale perspective, from the molecular to the anatomical realm, is essential for resolving the confounding relationships between structure and pain, elucidating the intricate mechanisms regulating joint health and diseases, and guiding the development of new treatment strategies. In this study, we developed the MUSIC method for 3D whole joint cellular and molecular mapping by resolving key challenges faced with joint immunostaining and tissue clearing and integrating with advanced large‐field light sheet imaging. The strength of our approach lies in its ability to collect comprehensive 3D information from entire joints and to generate multimodal data, including macroscale soft and hard tissue anatomy and microscale cellular and molecular patterns, on a single platform without the need for image coregistration. Existing tools cannot fully capture this level of detail. We demonstrated the power of our method by applying it to multiple joint systems (i.e., TMJs, knees, and spines) and various species (i.e., mice, rats, and pigs). Our results revealed heterogeneity in neurovascular structures within and across tissues, helping to explain joint complexity. We also found a similarity in overall neurovascular distribution between mice and rats, underscoring the conservation of joint organization. Furthermore, MUSIC enables the detection of biological features often missed with 2D approaches, such as nerve branching from main nerve bundles (Figures 1d and 4c) and innervation at the intersections of sacral segments (Figure S7c, Supporting Information). Together, these data uncovered the physical neurovascular connections among distinct joint components, supporting the whole‐joint concept^[^ ^4^ ^]^ and suggesting that changes in one tissue may directly influence other interconnected structures.

As a non‐invasive method that requires no sample sectioning, our method allows non‐skeptical and non‐biased data interpretation and more accurate data quantification, particularly for structures like nerves and vessels with complex and heterogeneous 3D networks, as shown in our data. Given that most existing studies on joint neurovascular structure are based on 2D histology studies and have reported the data as the nerve length or density in the plane of section (typically calculated as nerve length per unit area or as an area ratio),^[^ ^42^, ^46^, ^51^, ^52^ ^]^ it is challenging to have a direct comparison of our 3D results with the literature 2D results. Only a few studies reported neurovascular segment length density per tissue volume using 3D confocal imaging of thin (20 µm) dissected joint sections. These studies reported nerve and blood vessel length densities of ≈200–300 mm mm^−3^,^[^ ^53^ ^]^ which is within the same order of magnitude as our results in TMJ disc tissues (513 mm mm^−3^ in the sham group) (Figure 3f). In addition, our 3D mapping also recapitulates the spatial patterns and disease‐associated changes reported in the literature. For example, in the TMJ disc, we observed high nerve density in the anterior, posterior, and lateral regions, limited innervation on the medial side, and no innervation in the central region, consistent with previous reports.^[^ ^54^ ^]^ We also found increased nerve density of TMJ capsule tissues in the *Prg4* knockout joint degeneration model, which aligns with findings reported in other chemical‐induced joint degeneration models.^[^ ^51^, ^53^ ^]^ It is worth noting that literature reports on disease‐related changes of joint innervation based on the 2D method remain inconsistent across studies.^[^ ^42^, ^52^, ^55^ ^]^ Using our method, we aim to establish a quantitative and comprehensive database of the 3D cellular and molecular profile of the joint to advance mechanistic understanding and support translational and clinical research. This spatially resolved dataset can serve as a template for the field. For instance, the neurovascular information could facilitate the design of joint‐mimicking in vitro models.^[^ ^56^ ^]^

Our results showed neurovascular changes as joints degenerate and get injured, revealing the microscopic‐level structural responses at different disease stages that complement the macroscopic‐level changes, such as tissue morphology changes accessed through conventional imaging modalities (e.g., CT and MRI). Using the FMO mouse model, we demonstrated that microscale changes in joint innervation, rather than macroscale joint structural alterations, are correlated with pain behaviors. These findings provide direct evidence linking joint microstructure to pain, highlighting a potential structural basis for pain sensation in joint disease. This also suggests that the longstanding discrepancy between radiographic findings and clinical pain reports may arise from the absence of microscale information. Identifying new biomarkers based on these microscale structures may improve diagnostic accuracy and guide targeted therapies. Furthermore, integrating our high‐resolution data with advanced computational modeling offers the opportunity to link joint microstructure and macrostructure to biomechanics, function, and pain.^[^ ^57^ ^]^ Our mapping data contains anatomical and morphological information of hard and soft tissues and microscopic cellular and molecular information, an ideal foundation for developing an integrated computational modeling linking joint structure, function, and pain.

Our data reveal disease‐specific neurovascular changes in two complementary models of joint disease. Prior literature studies in both TMJ and knee have reported divergent neurovascular responses in diseases.^[^ ^42^, ^51^, ^55^ ^]^ When data from these two disease models are analyzed together, the relationship between joint microstructure and pain may not be immediately evident; however, clear correlations emerge when each disease model is analyzed individually. In the degenerative model, increased innervation correlates with pain, whereas in the injury model, decreased innervation is associated with pain, underscoring the disease‐specific and complex nature of pain mechanisms. In the joint degeneration model (*Prg4^‐/‐^*), pathology progresses over time, with both macroscale morphological changes and microscale neurovascular remodeling. The *Prg4^‐/‐^* joints exhibit marked inflammatory changes, including synovial hyperplasia, which may provide biochemical cues promoting ectopic nerve ingrowth and sprouting, as supported by our data and previous chemically induced joint degeneration studies.^[^ ^51^, ^53^ ^]^ Increased nerve density combined with pro‐inflammatory signaling likely contributes to heightened pain perception. By contrast, in the FMO model, macroscale joint morphology remains largely intact while microscale neurovascular alterations occur. Excessive mechanical loading of the TMJ disc during FMO procedures could cause nerve injury^[^ ^58^ ^]^ before detectable morphological changes. Elevated pain in FMO mice may thus reflect sensitization of remaining nerves or neuropathic‐like pain involving the trigeminal or central nervous system arising from nerve terminal injury.^[^ ^47^, ^49^ ^]^ Together, our findings provide quantitative evidence and mechanistic insight into pain across distinct disease contexts and may guide the development of targeted therapies. For example, strategies aimed at inhibiting aberrant nerve ingrowth may be beneficial in degenerative joint disease, whereas approaches focused on nerve repair or protection may be more appropriate for injury‐related pain.

Tissue clearing in the musculoskeletal field is in its infancy. To date, only a handful of tissue‐clearing protocols have been developed in the literature for single‐type mouse joint tissues,^[^ ^20^, ^21^, ^22^, ^23^, ^24^, ^25^ ^]^ and just one recently published protocol has reported whole‐joint clearing of the mouse knee with the use of collagenase, a method that carries the risk of compromising tissue structure.^[^ ^59^ ^]^ No studies have demonstrated its application to diverse tissue structures in various joint types across different species. Although the clearing of the entire mouse body has recently been reported, these studies are not focused on the joints.^[^ ^35^, ^60^ ^]^ Their performance on the joints has yet to be demonstrated, and their application is also limited to mice due to the high cost of whole‐body clearing and labeling for large animals. Our method specifically addresses this challenging task based on the unique structure and composition features of musculoskeletal joints and achieves a broad application in different types of joints across multiple animal species. In addition, our use of immunostaining, rather than reliance on endogenous fluorescent proteins in transgenic animals, provides the flexibility to simultaneously examine multiple cellular or molecular targets across species at reasonable cost, leveraging the large commercial availability of antibodies and probes.^[^ ^35^, ^37^, ^38^ ^]^ Using our approach, we can also detect and visualize the neurovascular structure inside the bone (Figure 2g; Figure S6, Supporting Information). While this current study provides limited analysis and discussion of nerves and vessels in bone, future work will further investigate and characterize the neurovascular architecture of the bone. Due to the limited availability of fresh human joint specimens, the application of our method in human samples has yet to be demonstrated here. Since the size of pig TMJ samples used in this study is much larger than adult human TMJ samples, we do not anticipate major issues applying our methods to human TMJ samples. The performance of our method on large human knees and spines will need further investigation.

3D imaging is becoming increasingly popular, given the tremendous, exciting information it can provide. Many 3D imaging platforms have been developed. Light sheet microscopes stand out with their capacity for a large field of view and high‐speed imaging as both benefit whole joint mapping. Compared to conventional confocal microscope imaging that detects fluorescence signal in a pixel‐based manner with a low quantum efficiency photomultiplier tube, the light sheet microscope uses a sheet of light illuminating a layer of sample and captures information from the entire layer at once using a highly sensitive camera, significantly increasing the 3D imaging speed and reducing photobleaching effects.^[^ ^61^ ^]^ The light sheet microscope used in this study is also equipped with a special lens that provides a large field of view and a dynamic scanning algorithm optimized for uniform illumination of large samples. This configuration enables imaging of sample volumes up to 53 cm^3^, encompassing a broad range of joint specimens. Moreover, open‐sourced and low‐cost light sheet microscopes have been developed over the years.^[^ ^62^, ^63^, ^64^, ^65^ ^]^ 3D whole joint mapping using our method is anticipated to become a routine research tool to boost future research advancements. Additionally, other multiplexed imaging modalities, such as fluorescence lifetime imaging^[^ ^66^ ^]^ and Raman spectroscopy,^[^ ^67^, ^68^ ^]^ can be integrated with our method to achieve whole joint mapping of various subtype cellular and molecular structures. Our method may also adapt recent developments of tissue clearing in live animals^[^ ^69^ ^]^ for in vivo studies of joint pathology. Due to the complexity of the joint and the proximity of joint components, our data analysis is primarily based on manual or semiautomatic, labor‐intensive methods for segmentation (hours to days of labor work for each dataset). Future research on developing artificial intelligence‐based automatic segmentation methods suitable for the whole joint mapping dataset is critical to streamlining the entire procedure and extracting more insightful and quantitative information.

In conclusion, we present a scalable and versatile tool to map the cellular and molecular profile in the context of entire joints of various types and species. Using two complementary mouse models of joint degeneration and traumatic injury, we demonstrate disease‐specific microscale neurovascular plasticity in joint disease. Our findings further show that the relationship between joint structure and pain is not universal, underscoring the complex and disease‐specific nature of pain mechanisms. This platform offers a powerful tool for multiscale 3D analysis, deepening our understanding of joint health and disease mechanisms and contributing to a new and effective treatment strategy.

## 4 Experimental Section

### Sample Preparation

*Prg4^‐/‐^ mouse samples*: Adult wild‐type and *Prg4^‐/‐^* C57BL/6 male or female mice (#025737, Jackson Laboratory, Bar Harbor, ME) aged between 6 and 12 months old were utilized in this study to investigate the neurovascular patterns in joints with apparent osteoarthritis‐like joint degeneration. Mice usage was approved by the Institutional Animal Care and Use Committee (IACUC) at Columbia University Irving Medical Center (AC‐AABP1553). After euthanasia, mouse skins were carefully dissected from the whole body without damaging major blood vessels and causing bleeding. Then, the whole mouse body was placed in 10% formalin for fixation at 4 °C for one day. This blood retention procedure is intended to retain as much blood inside the vessels as possible to enhance its fluorescence intensity with blood autofluorescence. Afterward, TMJs and knees were dissected and incubated in 10% formalin solution at 4 °C for another two days to ensure thorough joint fixation. Fixed TMJs and knees were washed in 1x phosphate buffer saline (PBS) at least three times before further processing.

*Forced mouth opening (FMO) mouse samples*: Adult C57BL/6 male or female mice (#000664, Jackson Laboratory, Bar Harbor, ME) aged between 8 and 12 weeks were used in this study with approval from the IACUC at the University of Maryland. The mice were anesthetized with isoflurane inhalation (3% for induction and 1.5% for maintenance) and kept in the isoflurane chamber during the procedure described in our previous protocol.^[^ ^34^ ^]^ FMO mice were injured with a customized mouth opener (0.017 in × 0.025 in rectangular wire) placed between the maxillary and mandibular incisors (Figure 3a) for 3 h per day for five consecutive days, while the Sham mice received no procedures. Following each procedure, both FMO and Sham mice were fed a soft diet (DietGel Recovery, ClearH2O, Westbrook, ME) and water gel (HydroGel, ClearH2O, Westbrook, ME). FMO mice were euthanized by transcardial perfusion with heparinized PBS followed by 4% paraformaldehyde in PBS at 12 days, 26 days, and 58 days after the final FMO procedure, respectively. Mice were randomly assigned to different groups. Sham mice were terminated at 12 days as reference control. Mouse heads were then harvested and washed with PBS three times for further processing.

*Rat samples*: Fresh rat joints were collected from Sprague Dawley rats at the conclusion of other IACUC‐approved research projects at the Medical University of South Carolina and Columbia University Irving Medical Center (AC‐AABG8555). Rats were terminated and perfused transcardially with heparinized PBS and 4% paraformaldehyde in PBS. Rat TMJs were harvested from adult male or female rats aged 25–26 weeks, while rat knees and spines were obtained from adult female rats aged 10–20 weeks. Rat joints were then fixed in 4% paraformaldehyde for one day and washed in PBS.

*Pig samples*: Fresh porcine heads (Yorkshire, 6 months) were obtained from a local abattoir and used within 12 h post‐mortem. The pig TMJ disc with attached surrounding tissues was carefully dissected from the pig head and immediately fixed in 10% formalin solution at 4 °C for three days and then washed with PBS three times before further processing.

### MUSIC Protocol

As the joints have bony components, fixed joints were first decalcified with 20% EDTA‐2Na (dissolved in diH_2_O, pH = 8.0) (BDH4616, VWR, Radnor, PA) for 3–10 days with daily solution change at 37 °C on a shaker. Complete joint decalcification was examined and confirmed with µCT imaging, showing uniform contrast throughout the joints. Then, joints were washed with 1x PBS three times and stored in PBS with 0.01% sodium azide at 4 °C until further processing. Decalcified joints were incubated in 10% CHAPS (10232, Cepham Life Sciences, Fulton, MD) in diH_2_O at 37 °C with gentle shaking for 7 days to deeply permeabilize the joint tissues. Joints were then dehydrated with graded methanol (154903, Sigma, St. Louis, MO) solutions (25%, 50%, 75%, 100%, 100%, mixed with diH_2_O) followed by delipidation treatment of dichloromethane (270997, Sigma, St. Louis, MO)/methanol mixture (v/v, 2:1) for 1–3 days until joints sink. Then, joints were bleached in chilled fresh 5% H_2_O_2_ (H1009, Sigma, St. Louis, MO) in methanol overnight at 4 °C and rehydrated with methanol series (75%, 50%, 25%, PBS) at room temperature (RT). Joints were washed in Triton X‐100 (T8787, Sigma, St. Louis, MO) /PBS mixture (v/v, 0.2% in 1x PBS) two times at RT. To loosen the joint extracellular matrix without significantly sacrificing the joint integrity, joints were incubated in a guanidine solution composed of 4 m guanidine hydrochloride (97061, VWR, Radnor, PA), 0.05 m sodium acetate (S7670, Sigma, St. Louis, MO) and 2% (w/v) Triton X‐100 in 1x PBS (pH = 6.0) at RT for 3 days to remove the proteoglycans while keeping the collagen backbones. Then, the permeabilized joints were blocked in a blocking solution containing 6% donkey serum (017‐000‐121, Jackson ImmunoResearch, West Grove, PA), 10% dimethylsulfoxide (DMSO, BDH1115, VWR, Radnor, PA), and 0.2% Triton X‐100 at 37 °C for 3 days, and incubated in primary antibodies (Table S1, Supporting Information for a full list of antibodies used in this study) in an immunostaining buffer solution containing 3% donkey serum, 10% CHAPS, 2% Triton X‐100, 10%DMSO, 1% glycine (G8898, Sigma, St. Louis, MO), 1% heptakis(2,6‐di‐O‐methyl)‐β‐cyclodextrin (77158, VWR, Radnor, PA) in 1x PBS at 37 °C for 3–7 days depending on the sample size. Joints were then washed 3–5 times in 1x PBS at 37 °C for 3 days followed by incubation in an immunostaining buffer solution with secondary Alexa fluorescent dye‐conjugated secondary antibodies, including Alexa Fluor 647 donkey anti‐goat IgG antibody (A‐21447, ThermoFisher, Waltham, MA), Alexa Fluor 647 donkey anti‐mouse IgG antibody (A‐31571, ThermoFisher, Waltham, MA), and Alexa Fluor 594 donkey anti‐rabbit IgG antibody (A‐31573, ThermoFisher, Waltham, MA) at 37 °C for 3–7 days. All secondary antibodies used 1:500 dilution. Joints were washed 3‐5 times in 1x PBS at 37 °C for 3 days. All immunostaining steps were performed with gentle shaking to facilitate antibody penetration and washout. After the completion of immunostaining, joints were dehydrated in methanol series at RT for one day, delipidated in 100% DCM solution at RT until joints sink, and cleared in dibenzyl ether (DBE, 108014, Sigma, St. Louis, MO) at RT for 1–2 days until joints became optically transparent (Figures 1, 4, 5; Figure S7, Supporting Information). Detailed protocols for different joints and species are available in Table S2 (Supporting Information).

### Antibody Verification and Compatibility Validation

Antibodies used in this study are listed in Table S1 (Supporting Information). Specificity through 2D immunostaining was first verified. Mouse and rat spinal cords and skin, and pig optical nerves were used to screen anti‐calcitonin gene‐related peptide (CGRP), CD31, and neurofilament 200 antibodies. Fresh samples were harvested from animals at the conclusion of other IACUC‐approved research projects, fixed with 10% formalin at 4 °C for two days, and then washed in 1x PBS. Then, fixed samples were incubated in sucrose solutions, snap frozen, and sectioned into 10–15 µm frozen sections with a cryostat (CM1510S, Leica Microsystem, Inc., Exton, PA). Tissue sections were then washed in PBS to remove the O.C.T. (optimum cutting temperature) compound, permeabilized with 0.1% Triton X‐100 in PBS, blocked with 10% donkey serum for 1 h, and immunostained with primary antibody solution at 4 °C overnight. Next, the slides were washed and incubated in a secondary antibody solution at room temperature for 2 h. Slides were washed, stained with a DAPI solution (R37606, ThermoFisher, Waltham, MA), and mounted with Fluoroshield (F6182, Sigma, St. Louis, MO) and a cover glass. Negative samples were defined as the sections only stained with the secondary antibody solution. Slides were imaged with a Leica TCS‐SP5 confocal microscope (Leica Microsystem, Inc., Exton, PA) with 20x and 40x objectives. DAPI signal was captured with a 405 nm laser and emission filter range of 380–450 nm; signals from Alexa Fluor 488 dye were imaged with a 488 nm laser and emission filter of 490–540 nm; signals from Alexa Fluor 647 dye were detected with a 633 nm laser and emission filter range of 665–695 nm.

Our MUSIC method includes methanol pretreatment for delipidation and bleaching, and uses methanol as the dehydration agent. An antibody validation experiment was performed as described in the literature to ensure the compatibility of the antibodies used in this study with methanol.^[^ ^37^ ^]^ Frozen sections were dehydrated and incubated in 100% methanol at room temperature for 3 h and then rehydrated and proceeded with the immunostaining procedure described above.

### Whole Joint Immunostaining Validation

To evaluate the performance of our method on deep and comprehensive 3D immunostaining, the whole joint using our protocol with a secondary antibody with Alexa Fluor 647 dye was first immunostained (Figure S2a, Supporting Information). Then, instead of processing the joints for tissue clearing, the joint was snap‐froze and cut the joint into 10–15 µm sections for 2D immunostaining. The sections were stained with the same primary antibody used in the whole joint staining, but used a different secondary antibody with Alexa Fluor 488 dye (Figure S2a, Supporting Information). Slides were finally imaged with a confocal microscope by acquiring fluorescence signals from both the first‐round 3D immunostaining (647 channel) and the second‐round 2D immunostaining (488 channel). As the antibody can directly and efficiently label tissue sections, the 2D immunostaining provides a ground truth to evaluate the efficiency of whole joint immunostaining. One expects to see overlap of the fluorescence signals from the whole joint 3D immunostaining (647 dye channel) and 2D section immunostaining (488 dye channel) for the desired whole joint 3D immunostaining.

### Light Sheet Imaging

Cleared whole joint samples were transferred to a non‐toxic ethyl‐3‐phenylprop‐2‐enoate (ECi, 76806, VWR, Radnor, PA) solution with a similar refractive index to DBE before imaging.^[^ ^70^ ^]^ Mice and rat joints were imaged with an Ultramicroscope II (Miltenyi Biotec, Germany) with a Super Plan configuration, which is equipped with a sCMOS camera (4.2 Megapixel, Andor Technology, UK), five excitation lasers (405, 488, 561, 639, 785 nm), tube lenses for post‐magnification adjustment (0.6x, 1x, 1.67x, and 2.5x), and three objective lenses with dipping caps specifically designed and optimized for large‐field light sheet imaging (1.1x/NA 0.1, 4x/NA 0.35, and 12x/NA 0.53). Cleared joints were mounted on the sample holder and immersed in the cuvette filled with ECi solution. 3D image stacks were acquired using the following laser and filter settings: 488 nm laser, emission 525/50 nm for autofluorescence signal; 561 nm laser, emission 595/40 nm for fluorescence signals from Alexa Fluor 594 dye; 639 nm laser, emission 680/30 nm for fluorescence signals from Alexa Fluor 647 dye. Whole joint mapping with mouse TMJs was imaged with the 4x objective at 1x magnification at a z‐step interval of 1.62 µm, matched with the in‐plane pixel dimension. In contrast, mouse knees, rat TMJs, and rat spines were imaged with the 4x objective at 0.6x magnification at a z‐step interval of 2.71 µm. Rat knees were imaged with the 1.1x objective at 1x magnification at a z‐step interval of 5–20 µm. 3D data acquisition in the regions of interest was collected with a 4x objective at 1.66x magnification or a 12x objective at 1x magnification at a z‐step interval of 1 µm.

Pig TMJ samples were imaged with an UltraMicroscope Blaze (Miltenyi Biotec, Germany) equipped with a sCMOS camera (4.2 Megapixel, Andor Technology, UK) and a special objective lens with dipping caps. Pig TMJ samples were also imaged in ECi solution. Mosaic acquisition was performed with a 4 × 2 tile scan at a 10% overlap to cover the entire sample. The pig TMJ was imaged with the 1.1x objective at 1x magnification at a z‐step interval of 8 µm throughout the full thickness of the sample. 3D image stacks were acquired using a 639 nm laser and an emission filter of 680/30 nm.

### 3D Mapping Data Reconstruction and Quantification

Three‐dimensional image stacks were saved in TIFF format for each channel separately. ImarisFile Converter (version 9.9, Bitplane, UK) was used to convert the 3D image data into Imaris file format for further data processing. The tile scan data with pig TMJ was stitched with Imaris Stitcher (version 9.9, Bitplane, UK). The decalcified bone region, soft tissues (e.g., cartilage and meniscus), and muscles were visualized using a 561 nm laser and an emission filter of 595/40 nm (Figure S3a,c, Supporting Information). Each joint tissue was manually segmented using the “Surface” function in Imaris, and a surface was eventually created to reconstruct its 3D geometry (Figure S3b,d, Supporting Information). For the TMJ condyle as well as the femur and tibia of the knee, the segmentation included both the bone and the overlying cartilage, which were rendered together as a single solid unit. The segmented surfaces were rendered with different colors and textures in Imaris for visualization. The reconstructed joint structure can be used as a mask to highlight the neurovascular structure of each joint tissue. Due to the relatively high autofluorescence from the muscles, they were not displayed in all the TMJ data for better visualization of the joint neurovascular structures. Vessels and nerves within the mouse TMJ disc were only displayed in the FMO data from the whole joint mapping (Figure 3e). Noise signals in the mapping data due to the presence of particles and antibody entrapments were removed based on their unique features of high brightness and spheric shape.

The neurovascular structure was quantified in the lateral capsule with the synovial tissue of the TMJ in the *Prg4^‐/‐^* data and the anterior region of the TMJ disc in the FMO data. For the quantification in *Prg4^‐/‐^* data, given the well‐known effect of *Prg4* knockout on the synovial tissues, the lateral capsule and synovial tissues were selected as our region of interest. The lateral capsule and synovial tissues were manually segmented at the superior joint cavity, which is resident near the junction of the TMJ disc, zygomatic arch, and the zygomaticomandibularis muscles, with a *z*‐depth of 100 µm (Figure S4a, Supporting Information). For the quantification of FMO data, given the potential mechanical stress in the anterior region during forced mouth opening,^[^ ^46^, ^47^ ^]^ the anterior region of the TMJ disc was selected as our region of interest. Using the condyle as a spatial reference, the anterior region of the disc is identifed over the anterior tip of the condyle and uses a cubic of 300 × 300 × 243 µm^3^ (185 × 185 × 150 voxels) to crop the region of interest for quantification (Figure S4c, Supporting Information). Nerve fibers and blood vessels were traced manually using the “Filament” function and the “AutoPath” method to measure the segment length and branch point. The blood vessels were also automatically segmented using the “Surface” function to determine the blood vessel volume (Figure S4b,d, Supporting Information). The selected capsule and disc volume was then quantified using the “Surface” function and automatic segmentation. The nerve and blood vessel length and branch point density, and blood vessel volume ratio were calculated by dividing the total segment length, total number of branch points, and total blood vessel volume to the corresponding tissue volume, respectively.

### Histology Analysis

Fixed mice TMJs were decalcified and prepared for paraffin or frozen‐embedded sections. Sagittal sections of the TMJ were collected and stained with Hematoxylin & Eosin (H&E). Bright‐field images were captured on a Keyence BZ‐X800E microscope (Keyence, Raleigh, NC).

### Micro‐Computed Tomography (µCT) Imaging and Quantification

Fixed mice TMJs from the *Prg4^‐/‐^* and FMO models were scanned with a Scanco µCT 40 device (Scanco Medical, Southeastern, PA) at a voxel resolution of 18 µm to capture the bone morphology. The µCT dataset of the bony structure of TMJs was then imported to Imaris (version 10.1, Bitplane, UK). The “Surface” function of Imaris was used to reconstruct the TMJ condyle head. According to the literature definition,^[^ ^71^ ^]^ the length and width of the condyle head were then determined by measuring the distance between the outermost points of the anterior, posterior, lateral, and medial of the condyle, respectively, using the “Measurement Points” function of Imaris.

### Statistical Analysis

A two‐sided *t*‐test was performed to evaluate the condyle head length and width differences and the nerve fiber density and blood vessel density differences between the wild‐type and knockout groups in the *Prg4^‐/‐^* model. One‐way ANOVA with Bonferroni post‐hoc test was used to examine the condyle head length and width differences and the nerve fiber density and blood vessel density differences among the experimental groups in the FMO model. Each joint corresponds to a single measurement. In some cases, both left and right joints from the same animal were imaged and analyzed, which leads to the joint number is different from the number of mice. The animal number was taken as a random factor for the analysis. All analyses were performed in SPSS (IBM SPSS Statistics, Version 28.0, IBM Corp., Armonk, NY). Significant differences were reported at *p*<0.05, with descriptive statistics reported as mean ± standard deviation.

## Conflict of Interest

Clemson University has submitted a provisional patent application to the U.S. Patent and Trademark Office pertaining to the method (Tissue clearing and imaging techniques for 3D whole joint mapping in musculoskeletal systems) and its associated utilities presented in this manuscript. H.Y., T.Y., P.C., and J.C. are named inventors of this patent application. The remaining authors declare no competing interests.

## Author Contributions

The manuscript was written with contributions from all authors. All authors have given approval to the final version of the manuscript. H.Y. conceived and directed the study. H.Y., T.Y., P.C., and J.C. developed the method for tissue clearing and imaging. H.Y., P.C., T.Y., M.K.C., M.C.E., and J.S.L. designed the experiments. P.C. and J.C. performed all the joint clearing and imaging experiments. P.C., J.C., A.S., R.G.H., B.K., I.A., S.R., I.T., B.J.D., S.W. and Y.S.K. performed the µCT imaging, joint histology, and quantitative measurements. P.C., B.K., J.H., I.T., J.T.H., and M.Y. prepared the animals. P.C., J.C., and H.Y. analyzed and interpreted the mapping data. P.C., J.C., and H.Y. drafted the manuscript. P.C., J.C., M.K.C., M.C.E., J.S.L., T.Y., and H.Y. critically reviewed and edited the manuscript. P.C. and J.C. contributed equally to this work.

## Supporting information



Supporting Information

Supplemental Movie 1‐15

## Data Availability

Raw image data of this study are available from the corresponding author upon reasonable request.
