# Supplementary material for: Multiscale 3D Whole Joint Cellular and Molecular Mapping Reveals Disease‐Specific Neurovascular Plasticity Underlying the Structure‐Pain Relationship
Source: Adv Sci (Weinh). 2025 Nov 6;13(1):e11226. doi: 10.1002/advs.202511226 (PMC12767019; doi:10.1002/advs.202511226)
Supplement: Supplementary file 1 — Supporting Information [file ADVS-13-e11226-s002.pdf]

Supporting Information

Multiscale 3D Whole Joint Cellular and Molecular Mapping Reveals Disease-Specific Neurovascular Plasticity underlying the Structure-Pain Relationship

Peng Chen, Jiaxin Chai, Abirami Soundararajan, R. Glenn Hepfer, Benjamin Kheyfets, Jiaxin Hu, Ishraq Alshanqiti, Swarnalakshmi Raman, Ikue Tosa, Jun Tae Huh, Matthew Yee, Brooke J. Damon, Shangping Wang, Yu Shin Kim, Man-Kyo Chung, Mildred C. Embree, Janice S. Lee, Tong Ye, Hai Yao**

Content

Figure S1-S7
Table S1-S2
Movie S1-S15

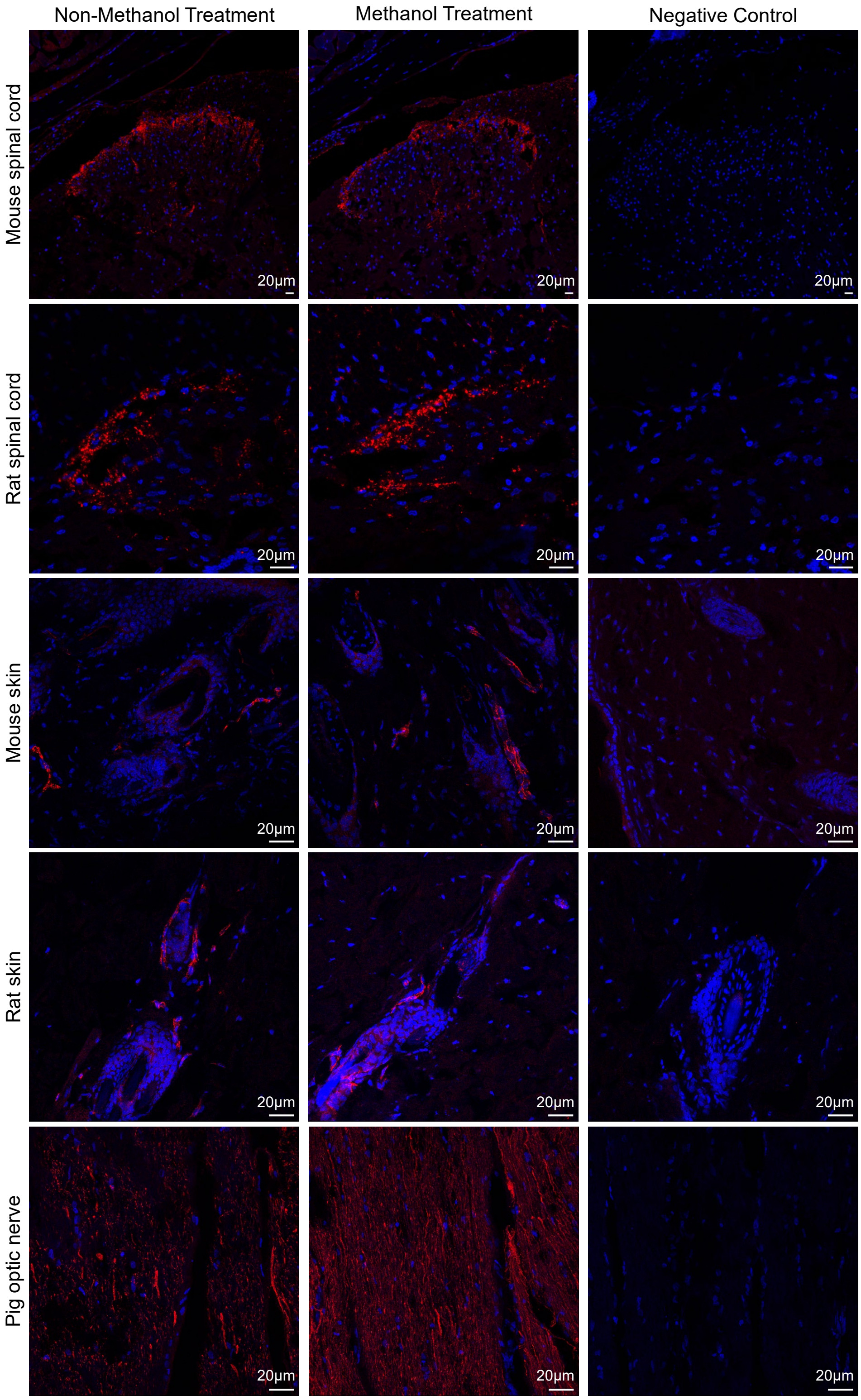

Figure S1. Antibody verification and compatibility with musculoskeletal joint immunostaining and clearing technique (MUSIC). The Goat anti-CGRP antibody was used to stain the frozen sections of the mouse spinal cord (see antibody information in **Table S1**). Our results showed confined CGRP immunostaining at the outer lamina of the spinal dorsal horn, agreeing with the literature reports. Methanol treatment does not affect the fluorescence intensity, validating the compatibility of this antibody for the MUSIC method. Mouse anti-CGRP antibody was applied to label the rat spinal cord frozen section. Similar results were seen with CGRP staining at the surface of the spinal dorsal horn, and no apparent fluorescence changes were observed after methanol treatment. Rabbit anti-CD31 antibody was utilized to

stain the mouse and rat skin. Vessels were observed in mouse and rat skin samples, and the same structures with similar fluorescence intensity were seen in the methanol treatment sections. Mouse anti-neurofilament 200 antibody was used to label pig optic nerve sections. Long nerve fibers running parallel to the optic nerve bundles were captured, and their fluorescence intensity was not affected after methanol treatment. All negative control samples showed no immunostaining signals. These results verified the specificity of the antibodies for the specific molecular targets and demonstrated their compatibility with the MUSIC method. Scale bar, 20 μm.

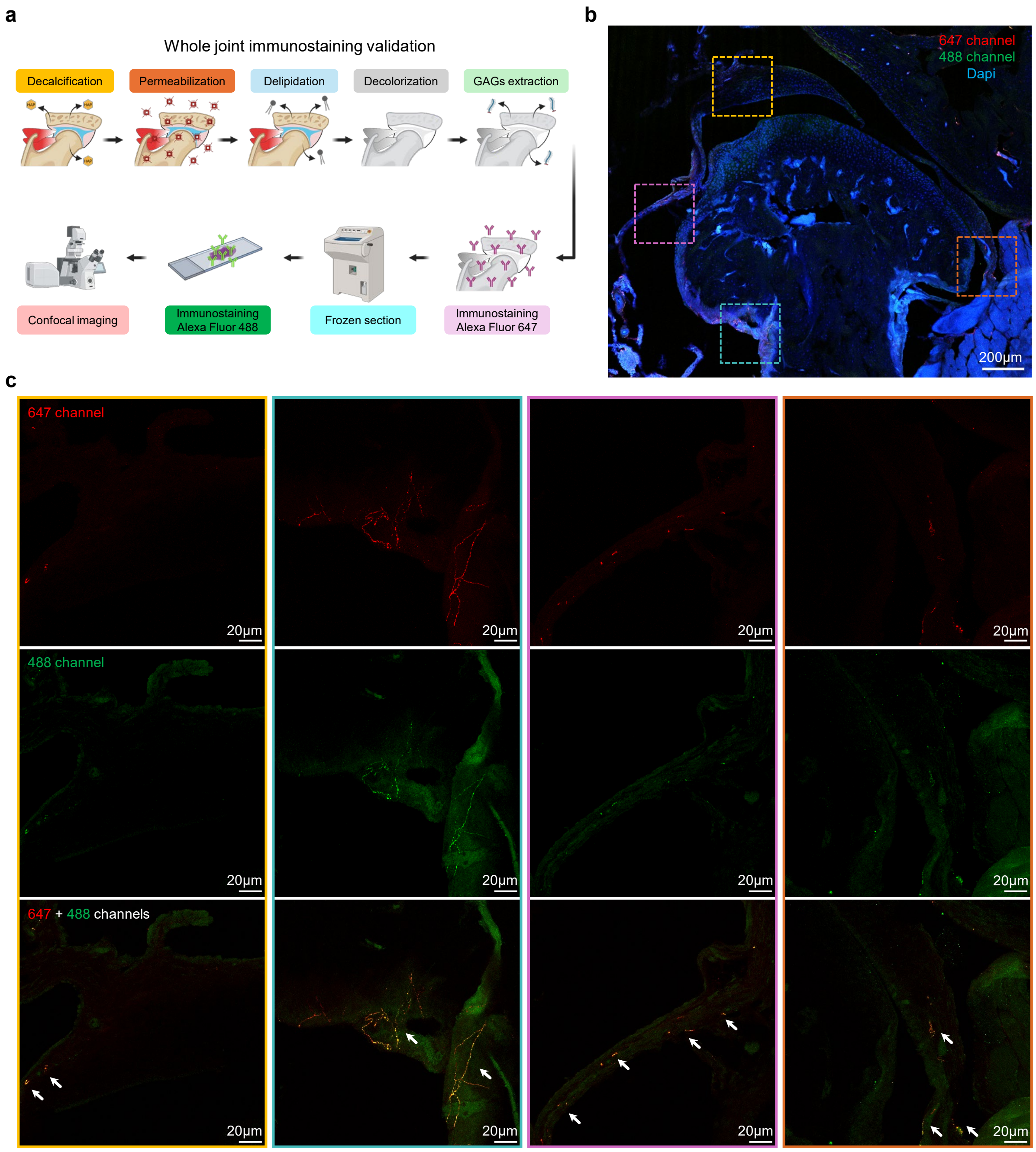

Figure S2. Whole joint immunostaining validation. a. Schematic of sample preparation for whole joint immunostaining validation. Mouse TMJs were processed following the MUSIC protocol for decalcification, permeabilization, delipidation, decolorization, and GAGs extraction. Then, whole TMJs were immunostained with primary antibodies for CGRP+ nerve fibers and secondary antibodies tagged with an Alexa Fluor 647 dye. Next, the immunostained joint was prepared for frozen section and cut into 10-15 µm sections and stained with the same primary antibody and a different secondary antibody with Alexa Fluor 488 dye. Joint sections were then imaged with a confocal microscope. **b.** A typical mouse TMJ section captured under the confocal microscope with a 20x objective. The dashed boxes highlight the regions for higher magnification imaging using a 40x objective. Scale bar, 20 µm. **c.** High-resolution imaging at the highlighted regions shown in panel **b**. Nerve fibers were detected in both channels, and their signals overlapped (white arrows), demonstrating that the whole joint immunostaining successfully labeled the target structures. Scale bar, 20 µm.

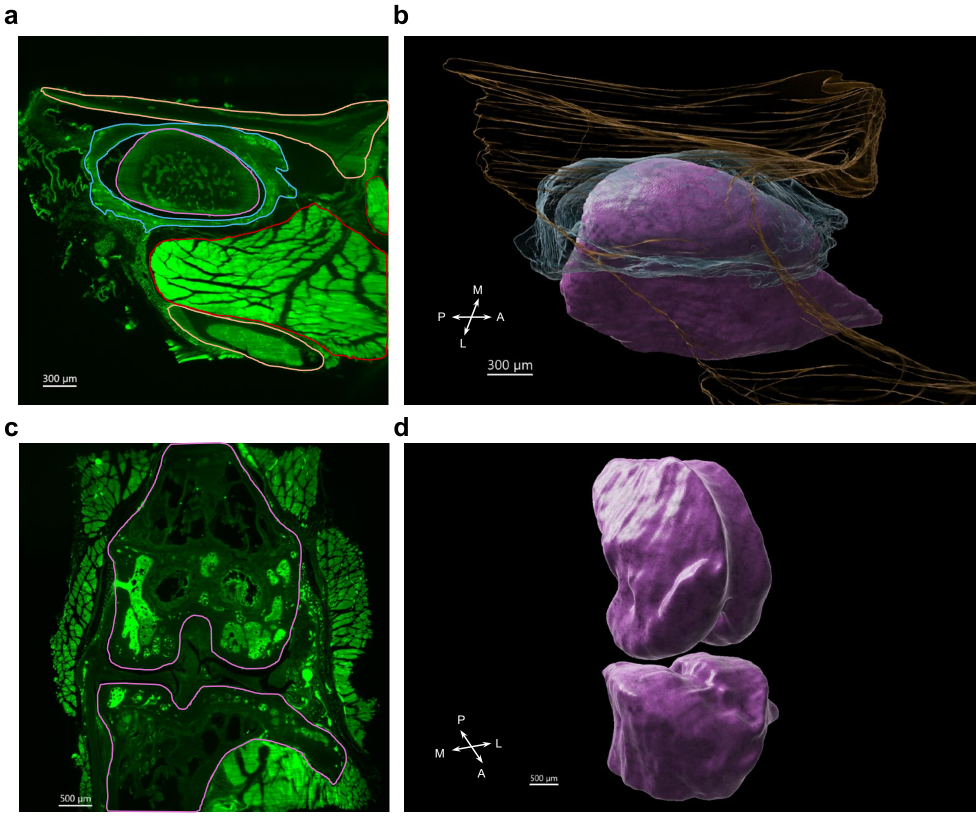

Figure S3. Joint mapping data and segmentation. a. Typical 2D optical section collected from the mouse TMJ mapping data. The image was acquired using a 561 nm laser and an emission filter of 595/40 nm. The image intensity was intentionally enhanced for visualization. Each joint component was manually segmented in Imaris using the "Surface" function. The TMJ condyle, including both the bone and overlying condylar cartilage, is circled in purple, the disc is highlighted in cyan, the fossa is shown in orange, and the muscle is depicted in red. A series of 2D optical sections from the whole joint mapping dataset were segmented to reconstruct the 3D geometry of joint components. Scale bar, 300 µm. **b.** 3D reconstruction of major joint components in mouse TMJ. The TMJ condyle, disc, and fossa are shown in the colors of purple, cyan, and orange, respectively. The 3D surface of joint components was rendered in Imaris for visualization. Scale bar, 300 µm. **c.** Typical 2D optical section in the mouse TMJ mapping data. The image was acquired using the same settings in **a**. The femur and tibia, including both the bone and overlying cartilage, are circled with purple lines. Scale bar, 500 µm. **d.** 3D reconstruction of femur and tibia in mouse knees. Scale bar, 500 µm. A, anterior; P, posterior; M, medial; L, lateral.

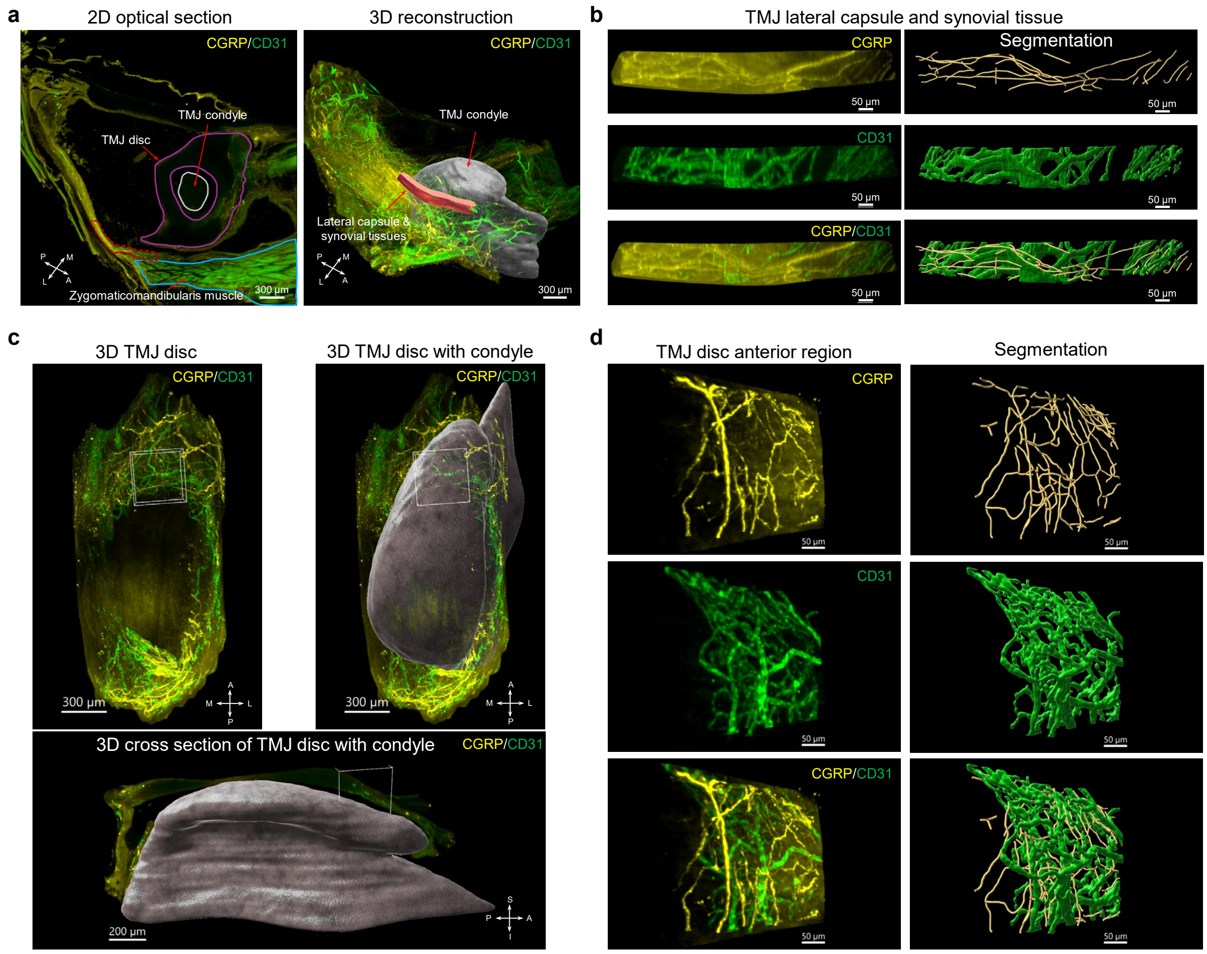

Figure S4. Joint mapping neurovascular structure quantification. a. 2D optical section and 3D view of neurovascular mapping in the whole mouse TMJ in *Prg4*$^{-/-}$ mice. The red dashed line highlights the segmented regions of the lateral capsule with synovial tissues. Red arrows indicate the TMJ condyle (including both the bone and overlying condylar cartilage) (outlined in grey), disc (outlined in purple), and zygomatico-mandibularis muscles (outlined in cyan). The 3D volume rendered in red color highlights the segmented regions of the lateral capsule with synovial tissues. Scale bar, 300 µm. **b.** 3D view of the nerves and blood vessels and their segmentations in the lateral capsule and synovial tissues. The length and branching point of nerves and blood vessels, and blood vessel volume were then quantified. Scale bar, 50 µm. **c.** 3D neurovascular structure in mouse TMJ disc displayed with and without condyle and the 3D cross section of the TMJ disc with condyle. A region of interest at the anterior region of the TMJ disc was selected, shown as the white cubic. Scale bar, 200 µm for the top images and 300 µm for the bottom image. **d.** 3D view of the nerve and blood vessel and their segmentations at the anterior region of the TMJ disc. The length and branching point of nerves and blood vessels, and blood vessel volume were then quantified. Scale bar, 50 µm. A, anterior; P, posterior; M, medial; L, lateral; S, superior; I, inferior.

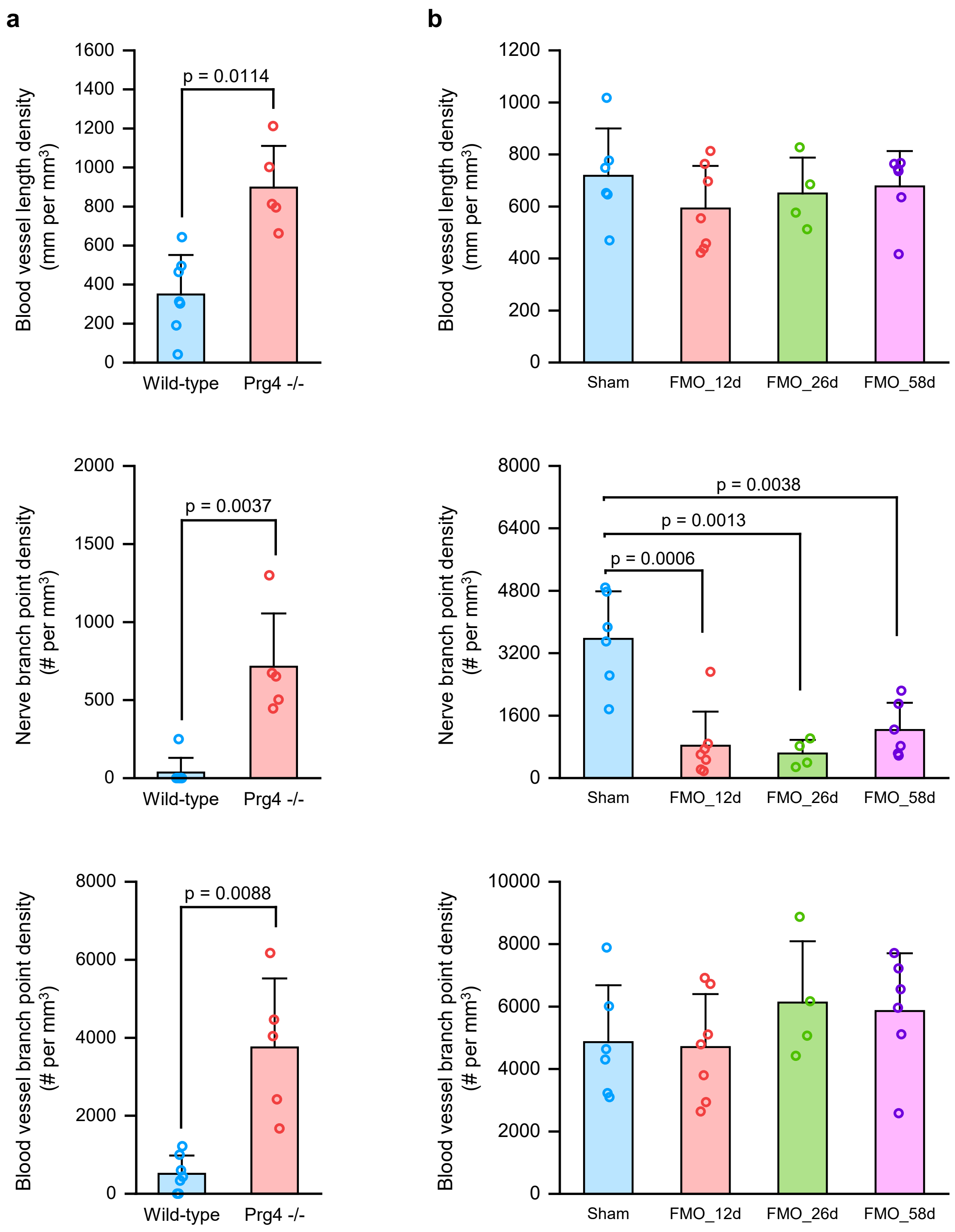

Figure S5. Neurovascular structure quantitative results. a. Quantitative blood vessel length density, and nerve and blood vessel branch point density in wild-type (n = 7 joints from 4 mice) and *Prg4*$^{-/-}$ TMJs (n = 5 joints from 3 mice). The nerve and vessel densities were quantified at the lateral capsule with synovial tissues. p-value was determined by a two-sided t-test. **b.** Quantitative blood vessel length density, and nerve and blood vessel branch point density in Sham (n = 6 joints from 4 mice), FMO_12d (n = 7 joints from 5 mice), FMO_26d (n = 4 joints from 2 mice), FMO_58d (n = 6 joints from 4 mice). The nerve and vessel densities were quantified at the anterior region of the TMJ disc. p-value was determined by one-way ANOVA with Bonferroni post-hoc test. All data depict mean ± standard deviation.

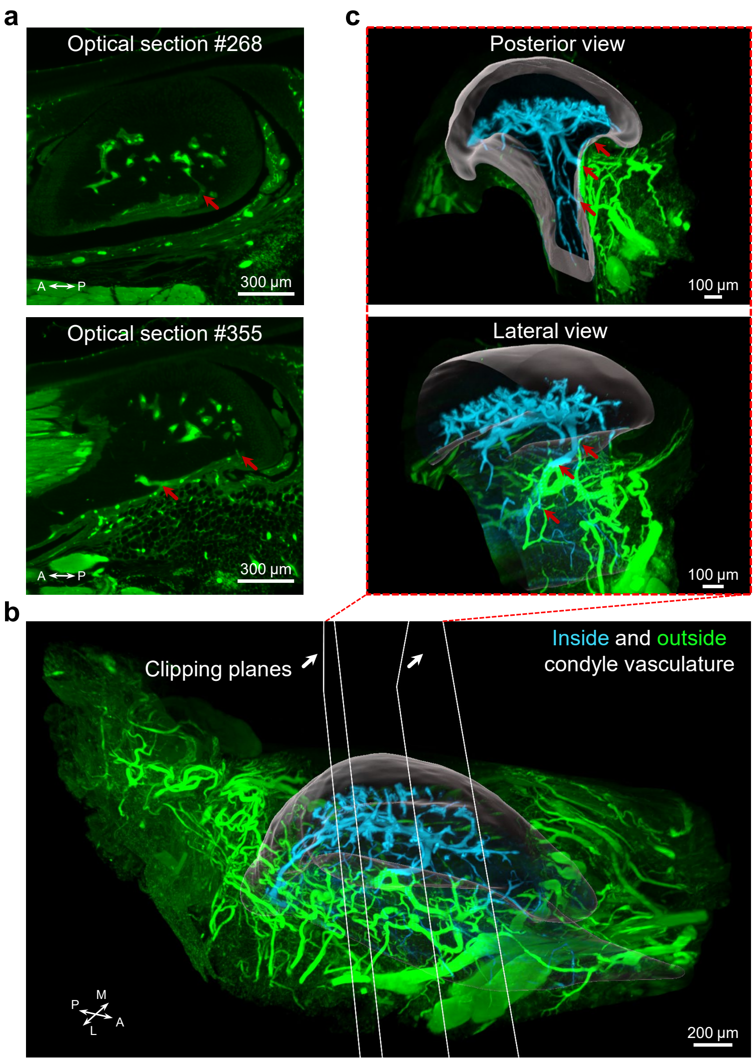

Figure S6. Vasculature connectivity inside and outside the TMJ condyle. a. Optical sections of 3D vasculature mapping data in a mouse TMJ. The vasculature channels that connect the inside and outside of the condyle are highlighted with red arrows. Scale bar, 300 µm. **b.** 3D reconstruction of the 3D vasculature structures in mouse TMJ. The vasculature inside and outside the condyle is labeled as cyan and green colors, respectively. The condyle, including both the bone and overlying condylar cartilage, was segmented and rendered as a transparent grey color. The clipping planes were added to crop the middle sections of the 3D dataset to show a zoom-in view of the vasculature connectivity, as shown in **c**. Scale bar, 200 µm. **c.** Posterior and lateral view of the cropped sections of the 3D whole TMJ vasculature dataset. Red arrows highlight the connectivity point between the inside and outside condyle vasculature. Scale bar, 100 µm. A, anterior; P, posterior; M, medial; L, lateral.

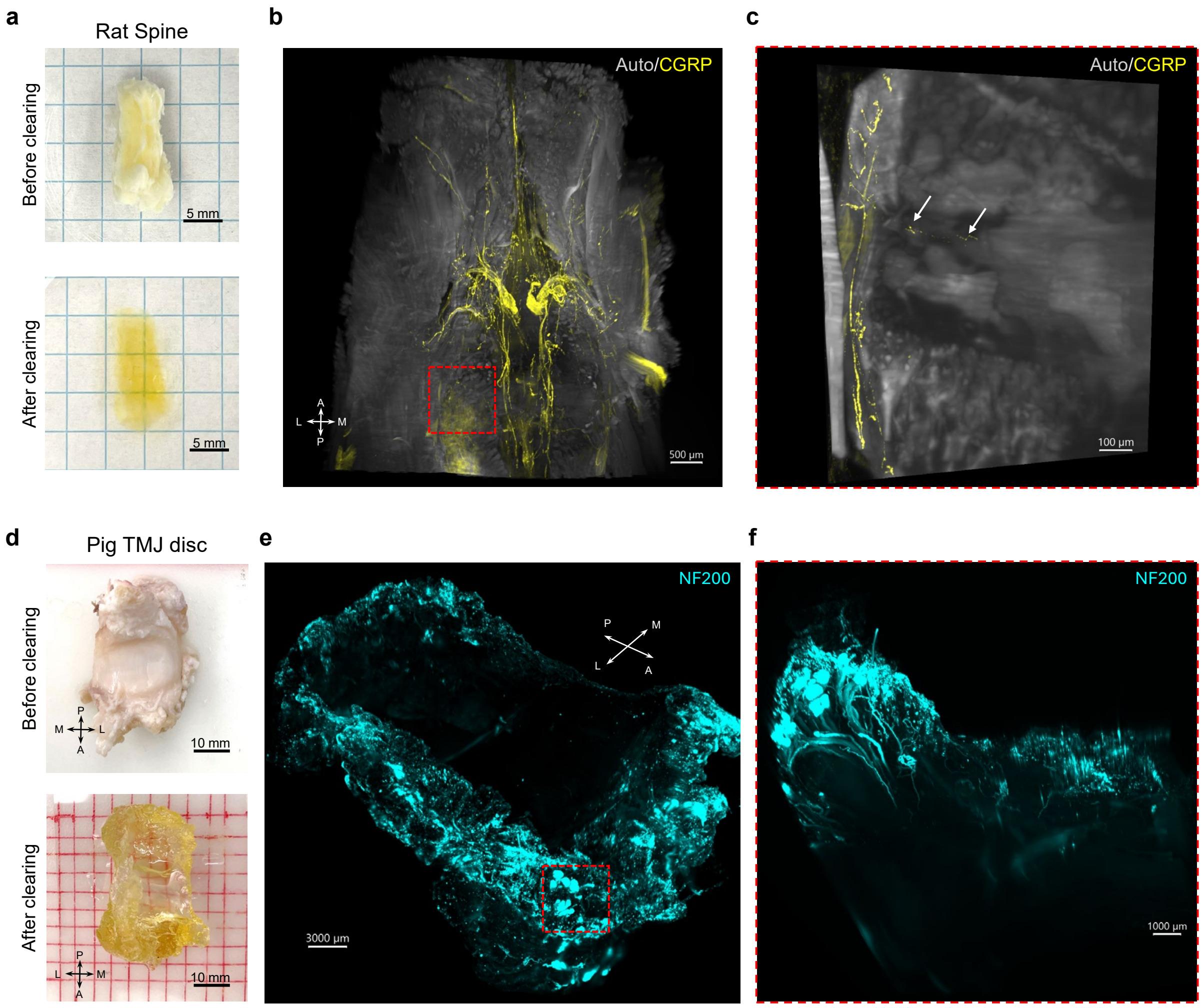

Figure S7. Whole joint mapping in large joint samples. a. Photos with rat spine samples before and after tissue clearing. Scale bar, 5 mm. **b.** Whole joint mapping results with CGRP stained rat spine near the sacrum sections. CGRP signals were captured using a 639 nm laser with an emission setting of 680/30 nm. Autofluorescence (Auto) was collected using a 488 nm laser with an emission filter of 525/50 nm. Scale bar, 500 µm. **c.** High magnification mapping data at the intersection of two sacrum vertebrae, dashed red square shown in panel **b**. CGRP nerve fibers (arrows) were seen innervating the bony region between two sacrum vertebrae. Scale bar, 100 µm. **d.** Photos with pig TMJ disc samples before and after tissue clearing. This pig sample also includes the surrounding soft tissues, such as the retrodiscal tissue. The sample size is approximately 33.5 mm × 43.8 mm × 15.6 mm. Scale bar, 10 mm. **e.** The neural mapping in the entire pig TMJ immunostained with NF200. The high density of neural staining was observed in the anterior, lateral, and posterior regions. Scale bar, 3000 µm. **f.** High magnification mapping data at the anterior-lateral region as highlighted in **e** with a dashed red square. Large nerve bundles and small nerve structures were detected. Scale bar, 1000 µm

Table S1: Primary antibodies used in the study and their dilutions.

Primary antibodies	Tested samples	Dilutions	Vendors	Cat No.
Goat anti-CGRP	Mouse TMJs and knees	1: 1000	Biorad	1720-9007
Mouse anti-CGRP	Rat knees and spines	1: 200	Abcam	ab81887
Mouse anti-NF200	Pig TMJs	1: 400	Sigma	N0142
Rabbit anti-CD31	Mouse TMJs and knees, rat TMJs	1: 50	ThermoFisher	PA5-16301

CGRP: calcitonin-gene related peptide
NF200: neurofilament 200
CD31: cluster of differentiation 31, or platelet endothelial cell adhesion molecule (PECAM-1)
TMJs: temporomandibular joints.

Table S2: Timeline of joint processing, immunostaining, and clearing.

Joints / Protocols	Mouse		Rat			Pig
	TMJs	Knees	TMJs	Knees	Spines	TMJs*
Decalcification	3	3	7	7-10	7-10	N/A
Permeabilization	7	7	7	7	7	7
Delipidation	1-2	1-2	1-3	1-3	1-3	2-3
Decolorization	1	1	1	1	1	1
GAGs extraction	3	3	3	3	3	3
Immunostaining	15	15	23	23	23	23
Clearing	1	1	2-3	2-3	2-3	2-3
Total time (days)	31-32	31-32	44-47	44-50	44-50	38-42

GAGs: glycosaminoglycans.

TMJs: temporomandibular joints.

N/A: not applicable.

*Pig TMJ samples contain only the TMJ disc and surrounding soft tissues, such as retrodiscal tissues, so the decalcification step was not applied.

Movie S1: 3D neurovascular mapping in mouse TMJ. The TMJ condyle, disc, and fossa were segmented and rendered in grey, translucent light blue, and translucent light brown, respectively.

Movie S2: 3D neurovascular mapping in wild-type mouse TMJ. The TMJ condyle was segmented and rendered in grey for spatial reference.

Movie S3: 3D neurovascular mapping in *Prg4*$^{-/-}$ mouse TMJ. The TMJ condyle was segmented and rendered in grey for spatial reference.

Movie S4: 3D vascular structures within a wild-type mouse TMJ condyle head. The TMJ condyle was segmented and rendered in translucent color for spatial reference.

Movie S5: 3D vascular structures within a *Prg4*$^{-/-}$ mouse TMJ condyle head. The TMJ condyle was segmented and rendered in translucent color for spatial reference.

Movie S6: 3D neurovascular mapping in Sham mouse TMJ. The TMJ condyles were segmented and rendered in grey for spatial reference.

Movie S7: 3D neurovascular mapping in FMO_12d mouse TMJ. The TMJ condyles were segmented and rendered in grey for spatial reference.

Movie S8: 3D neurovascular mapping in FMO_26d mouse TMJ. The TMJ condyles were segmented and rendered in grey for spatial reference.

Movie S9: 3D neurovascular mapping in FMO_58d mouse TMJ. The TMJ condyles were segmented and rendered in grey for spatial reference.

Movie S10: 3D neurovascular structures in wild-type mouse knee joint. The femur and tibia bones were segmented and rendered in dark grey for spatial reference.

Movie S11: 3D neurovascular structures in *Prg4*$^{-/-}$ mouse knee joint. The femur and tibia bones were segmented and rendered in dark grey for spatial reference.

Movie S12: 3D vascular structures in rat TMJ.

Movie S13: 3D neural structures in rat knee joint.

Movie S14: 3D neural structures in rat spine.

Movie S15: 3D neural structures in pig TMJ disc.
